# Best Time of Day for Strength and Endurance Training to Improve Health and Performance? A Systematic Review with Meta-analysis

**DOI:** 10.1186/s40798-023-00577-5

**Published:** 2023-05-19

**Authors:** Fabienne Bruggisser, Raphael Knaier, Ralf Roth, Wei Wang, Jingyi Qian, Frank A. J. L. Scheer

**Affiliations:** 1 Department of Sport, Exercise and Health, Faculty of Medicine, University of Basel, Basel, Switzerland; 2 Division of Sleep Medicine, Harvard Medical School, Boston, MA USA; 3 Medical Chronobiology Program, Division of Sleep and Circadian Disorders, Department of Medicine and Neurology, Brigham and Women’s Hospital, Boston, MA USA; 4 Division of Sleep and Circadian Disorders, Department of Medicine and Neurology, Brigham and Women’s Hospital, Boston, MA USA

**Keywords:** Circadian, Diurnal, Chronotype, Endurance, Strength, Performance, Cardiovascular, Metabolic, Systematic review, Meta-analysis

## Abstract

**Background:**

Current recommendations for physical exercise include information about the frequency, intensity, type, and duration of exercise. However, to date, there are no recommendations on what time of day one should exercise. The aim was to perform a systematic review with meta-analysis to investigate if the time of day of exercise training in intervention studies influences the degree of improvements in physical performance or health-related outcomes.

**Methods:**

The databases EMBASE, PubMed, Cochrane Library, and SPORTDiscus were searched from inception to January 2023. Eligibility criteria were that the studies conducted structured endurance and/or strength training with a minimum of two exercise sessions per week for at least 2 weeks and compared exercise training between at least two different times of the day using a randomized crossover or parallel group design.

**Results:**

From 14,125 screened articles, 26 articles were included in the systematic review of which seven were also included in the meta-analyses. Both the qualitative synthesis and the quantitative synthesis (i.e., meta-analysis) provide little evidence for or against the hypothesis that training at a specific time of day leads to more improvements in performance-related or health-related outcomes compared to other times. There was some evidence that there is a benefit when training and testing occur at the same time of day, mainly for performance-related outcomes. Overall, the risk of bias in most studies was high.

**Conclusions:**

The current state of research provides evidence neither for nor against a specific time of the day being more beneficial, but provides evidence for larger effects when there is congruency between training and testing times. This review provides recommendations to improve the design and execution of future studies on this topic.

*Registration*: PROSPERO (CRD42021246468).

**Supplementary Information:**

The online version contains supplementary material available at 10.1186/s40798-023-00577-5.

## Key points

- There is little evidence for or against the hypothesis that exercising at a certain time of day is more beneficial than exercising at another time of day in order to improve performance-related outcomes, anthropometrics, cardiovascular health outcomes, or cardiometabolic health outcomes.
- There is some evidence for a benefit of matching the time of day of training to the time of day of testing for performance-related outcomes.
- Because most studies show large heterogeneities, methodological limitations, and limited sample sizes, this review provides detailed recommendations in order to support performing high quality studies on this topic in the future.

## Introduction

Adherence to recommendations for physical activity leads to a reduction in cardiovascular diseases and reduction in premature mortality in diverse populations [1–5]. Current recommendations clearly define the frequency, intensity, type, and duration of physical activity. However, the time of day when the physical activity should be performed is not mentioned in any of the recommendations [6–8]. Various physiological functions are influenced by the time of day, such as core body temperature [9, 10], cardiovascular functions [11], respiratory control [12], endocrine factors [13–15], as well as subjective alertness [16], and thus might affect physiological adaptations in response to exercise. These variations are partly caused by the influence of the endogenous circadian system, causing endogenous circadian oscillations in biological processes following a cycle of approximately 24 h, i.e., that persist in the absence of environmental and behavioral cycles such as the dark/light, sleep/wake, and fasting/eating cycles [9, 12, 15–17]. Several reviews have discussed evidence for diurnal variations for many performance-related outcomes, with acrophases in the afternoon and evening [18–29]. A recent meta-analysis, for example, provided statistical evidence that mean power output in the 30-s Wingate test, jump height, as well as handgrip strength is higher in the late afternoon and early evening as compared to the morning [24]. The underlying mechanisms causing within-day and interindividual variations in peak performance are still unclear and may be due to myriad factors such as habitual exercise time, individual chronotype, sleep, food and caffeine intake, environmental conditions, and the endogenous circadian system [10]. Several reviews that investigated diurnal variations in maximum performance concluded that the time of day when the peak performance is achieved may also be the ideal timing for exercise [18–21, 29], with further reviews suggesting that the time of exercise training should coincide with the time of competition to achieve optimal performance improvements [22, 25, 30]. Observational studies found associations between the time of day of exercise and cardiorespiratory fitness [31], as well as the risk of coronary heart disease [31], obesity [32], prostate cancer, and breast cancer [33] suggesting that timing of exercise in fact might matter for improving performance and health outcomes in the long term. However, the results were inconclusive in regard to the ideal timing for exercise, with some studies suggesting morning exercise [32, 33] and some evening exercise [31, 33] to be more beneficial. Furthermore, these observational studies tested association and not causation. Therefore, longitudinal intervention trials are needed where the time of training is experimentally modified and differences in improvements are compared.

If timing of exercise training influences physical adaptations, this would have meaningful implications, because exercise recommendations would need to be updated to include the time of day and exercise intervention studies assessing changes in physical fitness and/or health-related outcomes would need to consider training and testing times. To date, ten reviews have discussed long-term effects of exercising during a specific time of day [19, 21, 22, 25, 30, 34–38]. However, eight of these are narrative reviews and thus are not based on a comprehensive systematic literature search and therefore reinforce subjective selection bias [19, 21, 22, 34–38]. The remaining two reviews [25, 30] conducted a systematic literature search. One review addressed primarily the topic of variation in maximal isometric and isokinetic performance throughout the day and included only a small proportion of studies that examined the long-term effects of exercise training at a specific time of day [25]. The other review was the only systematic review with meta-analysis and investigated the time-of-day specific effect of exercise training on muscle strength and muscle hypertrophy [30]. Thus, no systematic review to date has investigated the influence of exercise timing on health-related outcomes. In addition, the latter two reviews included non-randomized trials, which made the interpretation on causality unclear. They also only examined a narrow range of physical performance-related adaptations, and their approaches to assess risk of bias were somewhat lenient or not up to date [39]. Performing a comprehensive literature search and including an accurate assessment of the risk of bias are critical to interpreting the results. Therefore, the aim of this work was to perform a systematic review with meta-analysis to investigate if the time of day of exercise training in intervention studies influences physical performance, physical fitness, anthropometrics, cardiovascular or metabolic outcomes.

## Methods

This systematic review was conducted in accordance with the “Preferred Reporting Items for Systematic reviews and Meta-Analyses” (PRISMA) guidelines [40]. Eligibility criteria of the studies were: (1) including humans, (2) structured exercise interventions with a minimum of two exercise sessions per week and a minimum intervention duration of 2 weeks, (3) comparing physical exercise training interventions carried out at, a minimum of two different times of the day, and (4) implementing endurance and/or strength training interventions. Furthermore, (5) we only included randomized controlled trials, covering both cross-over designs where participants served as their own controls and were randomly allocated to a different sequence of interventions, and parallel group designs where participants were randomly allocated to one of different exercise intervention arms. There were no restrictions regarding date of publication and participants’ sex, age, fitness level, or health condition. Conference articles, literature reviews, and studies that were not written in the languages English, German, or French were excluded.

### Study Registration and Updates

The review was registered on March 31, 2021, on PROSPERO (CRD42021246468; https://www.crd.york.ac.uk/prospero/display_record.php?ID=CRD42021246468) and updated on January 10, 2022.

### Search and Study Selection

**Fig. 1:**
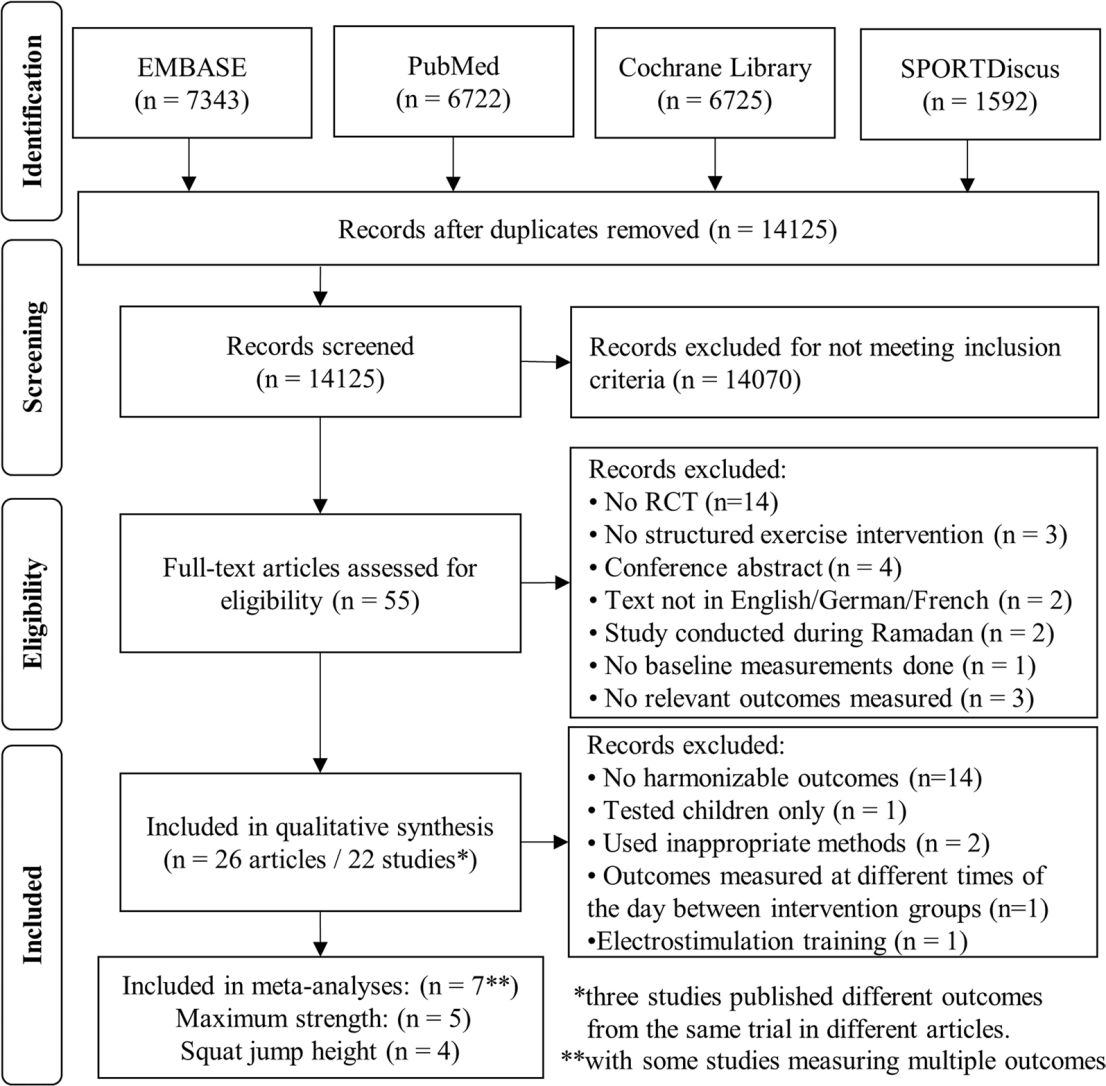
PRISMA study selection flow diagram

The initial search was conducted on May 11, 2021, and an updated search was conducted on January 4, 2023. For both searches, the databases EMBASE, PubMed, Cochrane Library, and SPORTDiscus were used (see Fig. 1). The four search strings were reviewed by an external data specialist. The complete search strings are contained in the Additional file 1: pages 1–7. The literature search and screening of all titles, abstracts, and full texts, in this order, for inclusion and exclusion of studies were performed by two independent reviewers (FB and RK). Results from these two independent screenings were compared, and disagreements between reviewers were resolved by mutual consensus and by involvement of a third expert (RR). To reduce the risk of screening fatigue, the two researchers conducted the screening of the studies in opposite order according to the alphabetical order of the family name of the first author. The full text of all articles that were reviewed for eligibility could be obtained.

### Data Collection Process and Data Items

The data extraction for each study that met all inclusion criteria was performed independently by two researchers (FB and RK). In cases of discrepancies, a third researcher was consulted (RR). The collected data included reference information, participant characteristics, the times of day when exercise was performed, the intervention duration, and frequency, duration, intensity, and type of the training sessions, as well as the outcomes. In addition, means, standard deviations, and other statistics were extracted for all outcomes. The details for all the extracted data are presented in Additional file 1: Table S1 (see pages 8–21). To estimate data from graphs for all studies for which the required data were not provided in tables or texts, the online tool web plot digitizer (https://automeris.io/WebPlotDigitizer/) was used.

### Risk of Bias Assessment

**Fig. 2:**
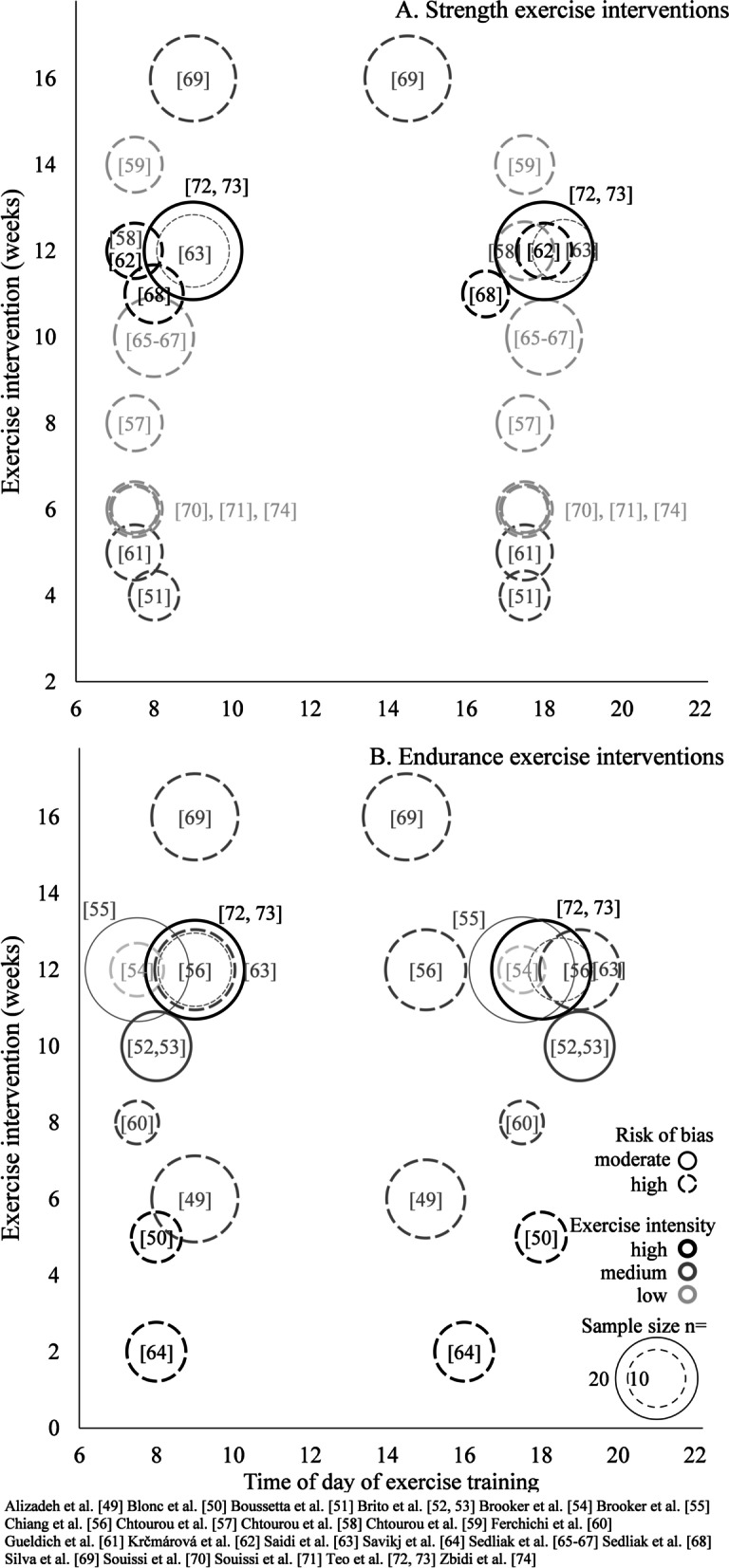
Description of the exercise interventions in the 22 studies included in the systematic review. Specific information is provided on training load (black/dark gray/light gray circle), type of exercise, time of day of exercise, and total duration of intervention. In addition, details on the sample size (size of the circle) and the existing risk of bias (open/closed circle) are presented for each study

The methodological quality of the included studies was assessed by two independent reviewers (FB and RK). In cases of discrepancies, additional researchers were consulted (RR, JQ, FAJLS). To evaluate the methodological quality of the included studies, a modified version of the “Cochrane risk of bias tool” [41] was used. The initial 22 criteria of the tool were transformed into 13 merged criteria relating to the topics of “bias arising from the randomization process,” “bias due to deviations from intended interventions,” “bias in measurement of the outcome,” “bias due to missing outcome data,” and "bias in selection of the reported result.” The criteria and the associated descriptions for assessing the risk of bias are provided in Table 1. All criteria were defined before the data extraction process started. For visualization purpose, the overall bias of each study was categorized as low, medium, or high risk of bias (see Fig. 2). Because most studies did not report effect sizes and 95% confidence intervals (see Additional file 1: Table S1—pages 8–21), the risk of bias across studies (i.e., publication bias) could not be assessed. Hence, funnel plots were created only for those studies included in the meta-analysis (see Additional file 1: Figure S1—page 22) using the same assumptions to calculate the standardized mean difference as for the meta-analyses.

**Table 1:** Risk of bias in individual studies

Study	1. Was the group allocation randomized?	2. Do baseline characteristics suggest a successful randomization?	3. Were participants blinded to their group allocation?	4. Were investigators blinded to participants’ group allocation?	5. Were gold-standard methods used to measure outcomes?	6. Were the methods used to measure outcomes appropriate?	7. Was an appropriate statistical analysis used?	8. Were data for outcomes available for most participants?	9. Were missing data similar between groups?	10. Were data for all outcomes reported?	11. Was a statistical correction for multiple outcomes performed?	12. Was an appropriate sample size calculation performed?	13. Was the study registered beforehand?	Overall risk of bias
Alizadeh et al. [49]	Yes	Yes	No	No	No	Yes	Yes	No	No	Yes	Yes	Yes	No	High (6)
Blonc et al. [50]^a^	Yes	Yes	No	No	No	Yes	Yes	Yes	NA	Yes	No	No	No	High (7)
Boussetta et al. [51]	Yes	Yes	No	No	No	No	Yes	Yes	NA	Yes	No	No	No	High (8)
Brito et al. [52, 53]	Yes	Yes	No	No	Yes	Yes	Yes	Yes	NA	Yes	Yes	No	Yes	Moderate (4)
Brooker et al. [54]	Yes	Yes	No	No	Yes	Yes	NA	Yes	NA	Yes	NA	No	Yes	High (6)
Brooker et al. [55]	Yes	Yes	No	No	Yes	Yes	Yes	Yes	NA	Yes	No	No	Yes	Moderate (4)
Chiang et al. [56]	Yes	Yes	No	No	No	No	Yes	Yes	NA	Yes	Yes	No	Yes	High (6)
Chtourou et al. [57]ª	Yes	Yes	No	No	No	No	Yes	Yes	NA	Yes	No	No	No	High (8)
Chtourou et al. [58]ª	Yes	Yes	No	No	No	Yes	Yes	Yes	NA	Yes	No	No	No	High (7)
Chtourou et al. [59]ª	Yes	Yes	No	No	No	Yes	Yes	Yes	NA	Yes	No	No	No	High (7)
Ferchichi et al. [60]	Yes	Yes	No	No	No	Yes	Yes	Yes	NA	Yes	No	No	No	High (7)
Gueldich et al. [61]	Yes	Yes	No	No	No	Yes	Yes	Yes	NA	Yes	No	No	No	High (7)
Krčmárová et al. [62]	Yes	Yes	No	No	No	No	Yes	Yes	NA	Yes	No	No	No	High (8)
Saidi et al. [63]	Yes	Yes	No	No	No	No	No	Yes	NA	Yes	No	No	No	High (8)
Savikj et al. [64]	Yes	Yes	No	No	No	No	Yes	Yes	NA	Yes	Yes	No	No	High (7)
Sedliak et al. [65–67]ª	Yes	Yes	No	No	No	Yes	Yes	No	Yes	Yes	No	No	No	High (7)
Sedliak et al. [68]	Yes	Yes	No	No	Yes	Yes	Yes	No	No	Yes	No	No	No	High (7)
Silva et al. [69]	Yes	Yes	No	No	No	No	Yes	Yes	NA	Yes	Yes	No	No	High (7)
Souissi et al. [70]ª	Yes	Yes	No	No	Yes	Yes	Yes	Yes	NA	Yes	No	No	No	High (6)
Souissi et al. [71]	Yes	Yes	No	No	No	Yes	Yes	Yes	NA	Yes	No	No	No	High (7)
Teo et al. [72, 73]	Yes	Yes	No	No	No	Yes	Yes	Yes	NA	Yes	Yes	Yes	Yes	Moderate (4)
Zbidi et al. [74]ª	Yes	Yes	No	No	No	Yes	Yes	Yes	NA	Yes	No	No	No	High (7)

Description indicating when a criterion has been answered with "yes": 5. gold-standard declarations: body composition = dual-energy x-ray absorptiometry, magnetic resonance tomography; jump height = force plate, infrared system; strength = isokinetic dynamometer, isometric dynamometer; glucose control = glucose clamp technique, intravenous glucose tolerance test. 6. appropriate methods in addition to the gold standard: body composition = bioelectrical impedance analysis, skinfold thickness; jump height = jump meter; strength = strain gauge, acceleration sensor; glucose control = oral glucose tolerance test, HbA1c (if exercise intervention is longer than 2 months). 8. cutoff: drop-out rate > 20%. 11. performing a statistical correction for multiple outcomes: definition of a primary outcome, valid sample size calculation for one outcome, trial registration with primary outcome. 12. performing an appropriate sample size calculation: precise sample size calculation with preliminary evidence for effect size. Overall risk of bias assessment: number of questions not answered with yes, 0–2 = low risk of bias, 3–5 moderate risk of bias, 6–8 high risk of bias, 9–11 very high risk of bias, 12–13 not included in this review due to inclusion criteria

ªIntervention studies included in the meta-analysis

### Synthesis of Results

**Fig. 3:**
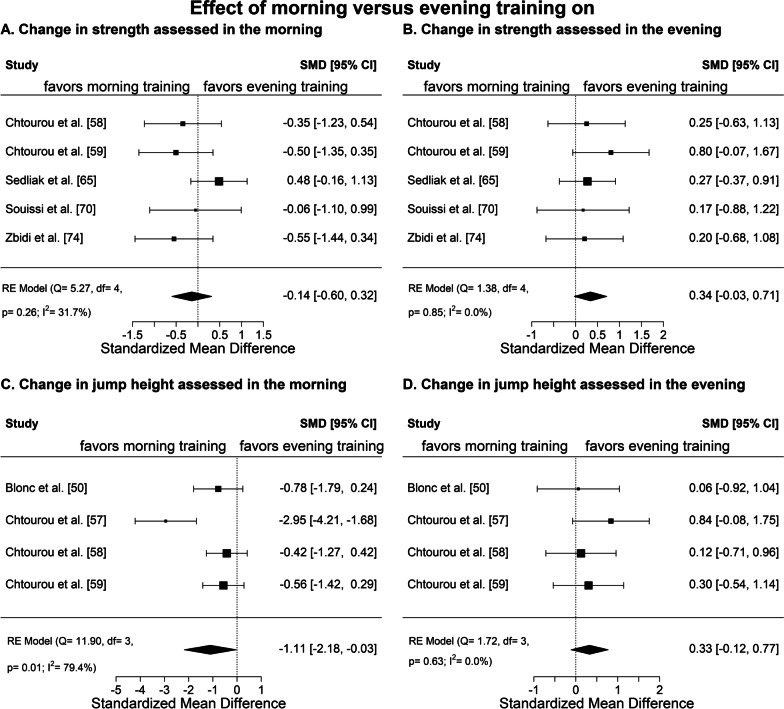
Meta-analysis of the standardized mean differences (SMD) for the change in performance due to the intervention, calculated as post-intervention value minus pre-intervention value, between morning training and evening training. Positive effect sizes indicate a higher improvement during the intervention period in the evening training condition, while negative effect sizes indicate a higher improvement in the morning training condition

**Fig. 4:**
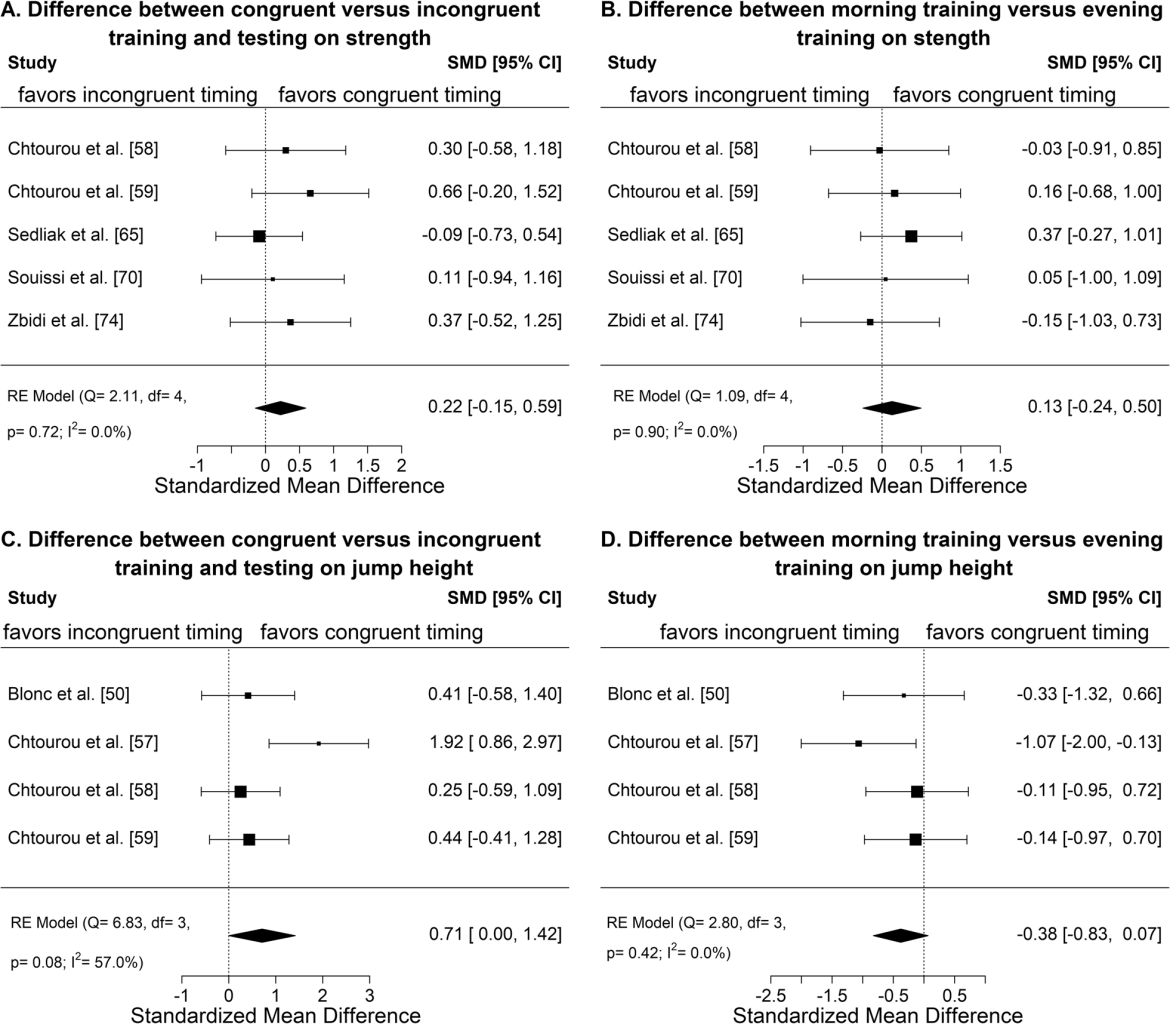
Meta-analysis of the standardized mean differences (SMD) for the change in performance due to the intervention (i.e., post-intervention value–pre-intervention value) between congruent training and testing in the morning and evening versus incongruent training and testing in the morning and evening for strength (**A**) and jump performance (**C**) and between morning exercise (tested both in the morning and evening) and evening exercise (tested both in the morning and evening) for strength (**B**) and jump performance (**D**). Positive effect sizes indicate a higher improvement during the intervention period in the evening training condition or with congruent training and testing, while negative effect sizes indicate a higher improvement in the morning training condition or with incongruent training and testing

The study characteristics of the 22 included studies are displayed graphically in Fig. 2, and the main results of these studies are reported in Table 2. Note that the specific outcomes examined in the meta-analysis are not presented therein to prevent overlap as they are shown in Figs. 3, 4, and Table S1, discussed below. Additional file 1: Table S1 (see pages 8–21) shows the detailed study characteristics and outcomes for all studies. Due to the low number of studies measuring harmonizable outcomes, a meta-analysis was only performed for maximum strength and jump height. The included seven studies for the meta-analysis used the gold standard method (i.e., isokinetic dynamometer and infrared system, respectively) or an appropriate method in addition to the gold standard (i.e., strain gauge and jump meter, respectively) to assess either outcome. When determining bilateral knee extension torque with the isokinetic dynamometer, different velocities and ranges of motion were used adding a layer of complexity. Thereby, participants were instructed to exert maximum voluntary effort with the fixed leg in both the concentric and eccentric phases of the movement pattern. The infrared jump system used in these studies to measure jump height was an Optojump photocell that emitted an infrared light 1–2 mm off the ground. During a squat jump or countermovement jump, the infrared light was disrupted by the participant's feet, causing the device to trigger a timer that could measure flight time and contact time. Due to limited comparability with the main group of studies, those studies that examined children, performed a one-repetition maximum test, or implemented electrostimulation training as an exercise intervention were excluded from the meta-analysis. Because effect sizes were not reported for the individual studies, we computed Cohen’s *d* to estimate the effect sizes [42, 43] for the individual studies included in the meta-analysis. Mean_AM_ ± SD_AM_ and Mean_PM_ ± SD_PM_ were defined as the group averages and the standard deviations of the change in the outcome from baseline to the end of the intervention period for the morning training condition and the evening training condition, respectively. To calculate the standard deviations of the change as they were not reported in the individual studies, we assumed a correlation of 0.5 between the baseline and post-intervention values. Effect size of the difference between the two conditions (AM vs. PM training) was defined as the change in outcome from the evening training condition minus the change in outcome from the morning training condition. Thus, positive effect sizes indicate a higher improvement during the intervention period in the evening training condition, whereas negative effect sizes indicate higher improvements during the intervention period in the morning training condition. Because the risk of bias was similar for all studies included in the meta-analysis, no correction was applied to adjust for study quality. However, studies were weighted by sample size with larger sample size giving more weight. A random-effects model was applied to estimate overall Cohen’s *d* using calculated Cohen’s *d* from individual studies. Figure 3 shows the forest plots that were used to display and compare estimates across studies. Heterogeneity among studies was estimated by the Cochran Q test and quantified by the *I*^2^ statistic with the respective *p*-value [44]. Because the studies included in the meta-analyses were similar regarding age, sex, fitness level, and health status, no sensitivity or sub-group analyses were performed.

**Table 2:** Summary of the main outcomes of individual studies

Study	Time of assessment			Pre-intervention						Post-intervention						Post–pre									
				Morning training			Evening training			Morning training			Evening training			Morning training			Evening training			(∆ evening training–∆ morning training)			(*p*)
				(mean ± SD)						(mean ± SD)						(mean difference)									
Anthropometric outcome measures																									
Body fat content (%)																									
Alizadeh et al. [49]	AM			35.7 ± 1.7			35.7 ± 2.1			34.6 ± 2.0			35.1 ± 2.3			− 1.1			− 0.6			0.5			0.26
Brooker et al. [54]	AM			41.7 ± 8.1			40.6 ± 8.9			40.5 ± 9.8			40.0 ± 8.3			− 1.2			− 0.6			0.6			n.r
Brooker et al. [55]	n.r			41.3 ± 7.4			42.7 ± 7.3			40.6 ± 8.1			41.6 ± 7.6			− 0.7			− 1.1			− 0.4			0.48
Krčmárová et al. [62]	AM			41.8 ± 4.9			42.2 ± 6.8			39.5 ± 5.7			40.4 ± 7.0			− 2.3			− 1.8			0.5			≥ 0.05
Saidi et al. [63]	AM			42.4 ± 7.8			42.1 ± 8.0			40.7 ± 7.9			39.6 ± 8.6			− 1.7			− 2.5			− 0.8			0.35
Quadriceps femoris volume (cm^3^)																									
Sedliak et al. [67]	n.r			2180 ± 340			2118 ± 217			2237 ± 342			2192 ± 220			57			74			17			0.19
Quadriceps femoris cross-sectional area (mm^2^)																									
Sedliak et al. [68]	AM/PM			7721 ± 795			8689 ± 1519			8385 ± 828			9674 ± 1495			664			985			321			≥ 0.05
Performance-related outcome measures																									
VO_2_max (mL/kg/min)																									
Brito et al. [52]	AM			21.4 ± 3.2			21.4 ± 3.4			23.1 ± 3.4			23.0 ± 4.6			1.7			1.6			− 0.1			≥ 0.05
	PM			22.2 ± 3.2			21.0 ± 4.1			24.5 ± 3.9			23.3 ± 3.8			2.3			2.3			0			≥ 0.05
Brooker et al. [54]	AM			30.7 ± 5.1			28.5 ± 7.0			35.9 ± 8.3			33.1 ± 8.7			5.2			4.6			− 0.6			n.r
Brooker et al. [55]	n.r			29.1 ± 6.3			28.0 ± 7.5			33.6 ± 8.2			33.2 ± 8.6			4.5			5.2			0.7			0.82
12.5 m swim performance (s)																									
Ferchichi et al. [60]	AM			9.66 ± 0.49			9.87 ± 0.75			7.37 ± 0.30			8.75 ± 0.21			− 2.29			− 1.12			1.17			** < 0.01**
	PM			7.81 ± 0.75			8.26 ± 0.87			7.43 ± 0.54			7.11 ± 0.14			− 0.38			− 1.15			− 0.77			** < 0.01**
30-s Wingate mean power output (W/kg)																									
Boussetta et al. [51]	AM		7.92 ± 0.17			8.51 ± 0.13			9.12 ± 0.24			8.39 ± 0.15			1.20			− 0.12			− 1.32				≥ 0.05
	PM		8.53 ± 0.13			9.14 ± 0.16			9.14 ± 0.20			9.11 ± 0.20			0.61			− 0.03			− 0.64				≥ 0.05
Chtourou et al. [57]	AM		7.93 ± 0.47			7.65 ± 0.58			8.87 ± 0.65			7.70 ± 0.58			0.94			0.05			− 0.89				** < 0.001**
	PM		8.21 ± 0.53			8.07 ± 0.49			8.76 ± 0.61			8.77 ± 0.55			0.55			0.70			0.15				** < 0.001**
Chtourou et al. [58]	AM		8.21 ± 0.95			8.31 ± 0.74			8.42 ± 0.81			8.50 ± 0.80			0.21			0.19			− 0.02				≥ 0.05
	PM		8.58 ± 0.76			8.76 ± 0.77			8.58 ± 0.83			9.00 ± 0.78			0.00			0.24			0.24				≥ 0.05
Souissi et al. [71]	AM		7.12 ± 0.64			7.01 ± 0.85			7.63 ± 0.68			7.22 ± 1.00			0.51			0.21			− 0.30				** < 0.05**
	PM		7.48 ± 0.59			7.48 ± 0.97			7.61 ± 0.51			7.98 ± 1.06			0.13			0.50			0.37				** < 0.05**
Electromyography activation (µV)																									
Gueldich et al. [61]	AM		970 ± 150			1040 ± 130			1600 ± 140			1630 ± 60			630			590			− 40				** < 0.01**
	PM		1340 ± 120			1270 ± 130			1670 ± 100			2120 ± 30			330			850			520				** < 0.01**
Sedliak et al. [66]	AM		324 ± 133			339 ± 153			379 ± 151			373 ± 157			55			34			− 21				≥ 0.05
	PM		321 ± 125			339 ± 159			369 ± 148			347 ± 177			48			8			− 40				≥ 0.05
Health-related outcome measures																									
FEV1 (L)																									
Silva et al. [69]	n.r	1.73 ± 0.29			1.74 ± 0.38			1.81 ± 0.24			1.80 ± 0.34			0.08			0.06			− 0.02					≥ 0.05
Resting systolic blood pressure (mmHg)																									
Brito et al. [52]	AM	133 ± 14			129 ± 10			128 ± 13			123 ± 5			− 5			− 6			− 1					** < 0.05**
	PM	134 ± 14			134 ± 11			134 ± 14			125 ± 7			0			− 9			− 9					** < 0.05**
Brooker et al. [54]	AM	119 ± 10			135 ± 6			114 ± 7			121 ± 8			− 5			− 14			− 9					n.r
Brooker et al. [55]	n.r	120 ± 16			126 ± 16			118 ± 15			120 ± 10			− 2			− 6			− 4					0.25
Resting diastolic blood pressure (mmHg)																									
Brito et al. [52]	AM	90 ± 6			91 ± 5			90 ± 6			87 ± 5			0			− 4			− 4					≥ 0.05
	PM	91 ± 6			92 ± 7			91 ± 7			89 ± 7			0			− 3			− 3					≥ 0.05
Brooker et al. [54]	AM	84 ± 8			86 ± 9			80 ± 6			84 ± 10			− 4			− 2			2					n.r
Brooker et al. [55]	n.r	85 ± 11			85 ± 11			81 ± 11			82 ± 9			− 4			− 3			1					0.08
Fasting glucose (mmol/L)																									
Brooker et al. [54]	AM	5.6 ± 0.7			6.0 ± 1.0			5.5 ± 0.5			5.3 ± 0.4			− 0.1			− 0.7			− 0.6					n.r
Brooker et al. [55]	n.r	5.5 ± 0.5			5.7 ± 0.5			5.5 ± 0.6			5.6 ± 0.5			0			− 0.1			− 0.1					0.41
Krčmárová et al. [62]	AM	5.8 ± 0.4			5.6 ± 0.9			5.6 ± 0.6			5.1 ± 0.5			− 0.2			− 0.5			− 0.3					** < 0.05**
Savikj et al. [64]	AM	7.3 ± 1.0			7.3 ± 1.0			7.7 ± 1.3			7.5 ± 1.0			0.4			0.2			− 0.2					≥ 0.05
Teo et al. [72]	AM	7.7 ± 1.7			8.3 ± 3.7			6.8 ± 1.5			7.1 ± 2.4			− 0.9			− 1.2			− 0.3					0.42
Hb1Ac (%)																									
Savikj et al. [64]	AM	6.6 ± 1.3			6.6 ± 1.3			6.3 ± 0.7			6.4 ± 0.7			− 0.3			− 0.2			0.1					≥ 0.05
Teo et al. [72]	AM	6.9 ± 1.2			6.8 ± 1.7			6.6 ± 1.1			6.5 ± 1.5			− 0.3			− 0.3			0					0.79
Insulin (pmol/L)																									
Savikj et al. [64]	AM	56.9 ± 30.1			56.9 ± 30.1			71.4 ± 22.9			70.4 ± 38.5			14.5			13.5			− 1					≥ 0.05
Teo et al. [72]	AM	88.3 ± 33.9			81.0 ± 29.7			64.4 ± 23.4			58.6 ± 22.1			− 23.9			− 22.4			1.5					0.85
Low-density lipoprotein (mmol/L)																									
Brooker et al. [54]	AM	3.1 ± 0.7			2.6 ± 0.6			2.7 ± 0.6			2.4 ± 04			− 0.4			− 0.2			0.2					n.r
Brooker et al. [55]	n.r	2.9 ± 0.8			2.7 ± 0.7			2.6 ± 0.7			2.8 ± 0.9			− 0.3			0.1			0.4					0.51
Krčmárová et al. [62]	AM	2.6 ± 0.8			3.4 ± 1.3			3.0 ± 0.9			3.6 ± 1.4			0.4			0.2			− 0.2					≥ 0.05
Savikj et al. [64]	AM	2.4 ± 1.3			2.4 ± 1.3			2.4 ± 1.3			2.3 ± 1.3			0.0			− 0.1			− 0.1					≥ 0.05
High-density lipoprotein (mmol/L)																									
Brooker et al. [54]	AM	1.7 ± 0.4			1.0 ± 0.1			1.5 ± 0.4			0.9 ± 0.1			− 0.2			− 0.1			0.1					n.r
Brooker et al. [55]	n.r	1.4 ± 0.5			1.3 ± 0.4			1.4 ± 0.4			1.3 ± 0.4			0			0			0					0.87
Krčmárová et al. [62]	AM	1.5 ± 0.5			1.5 ± 0.4			1.5 ± 0.4			1.7 ± 0.4			0.0			0.2			0.2					≥ 0.05
Savikj et al. [64]	AM	1.2 ± 0.3			1.2 ± 0.3			1.3 ± 0.3			1.2 ± 0.3			0.1			0.0			− 0.1					≥ 0.05
Triglycerides (mmol/L)																									
Brooker et al. [54]	AM	1.3 ± 0.8			1.4 ± 1.1			1.0 ± 0.4			1.2 ± 0.5			− 0.3			− 0.2			0.1 n.r					
Brooker et al. [55]	n.r	1.3 ± 0.7			1.4 ± 0.6			1.3 ± 0.7			1.4 ± 0.8			0			0			0					0.91
Krčmárová et al. [62]	AM	1.4 ± 0.4			1.7 ± 1.0			1.6 ± 0.4			1.3 ± 0.7			0.2			− 0.4			− 0.6					** < 0.01**

Note that the specific outcomes examined in the meta-analysis are not presented in the table to prevent overlap as they are shown in Fig. 3 and Table S1

*SD* standard deviation, *AM* at morning, *PM* past morning, *n.r.* not reported, *VO*_*2*_*max* maximal oxygen consumption, *FEV1* forced expiratory volume in one second, *HbA1c* hemoglobin A1c. All blood samples were collected in a fasted state

For all outcomes with a *p* ≥ 0.05, no specific *p* value was reported in the respective study

We also conducted a second meta-analysis. This second meta-analysis was a non-pre-specified exploratory meta-analysis (see Fig. 4) to compare temporally congruent testing and training with temporally incongruent testing and training (Fig. 4A, C) as well as to compare morning exercise and evening exercise considering both testing in the morning and evening (Fig. 4B, D). This is because (1) physical performance and many health-related outcomes exhibit diurnal variations [24, 45, 46], and (2) exercise has been shown to shift the rhythms in skeletal muscle clock and other metabolic pathways [47, 48]. Therefore, the assessed physical adaptations to training program may be affected by the relative timing of testing and training. Here we define congruent as exercise training and outcome testing taking place at the same time of day, i.e. evening testing in the evening training condition and morning testing in the morning training condition. Incongruent was thus defined as evening testing in the morning training condition and morning testing in the evening training condition. The same assumptions and methods were used for the exploratory meta-analysis as for the first/primary meta-analysis. However, for the second meta-analysis, Mean_1_ was calculated as the average of the deltas (post-intervention value minus baseline value) of the two congruent tests and Mean_2_ as the average of the deltas of the two incongruent tests. The positive effect sizes indicate a stronger improvement for congruent training and testing as compared to incongruent training and testing. For Fig. 4B, D Mean_1_ was calculated as the average of the deltas (post-intervention value minus baseline value) of the two tests (morning and evening testing) performed in the evening exercise group and Mean_2_ as the average of the deltas of the test in the morning exercise group. The positive effect sizes indicate a stronger improvement for evening exercise as compared to morning exercise.

## Results

### Study Selection and Characteristics

An overall view of the selected studies is presented in Fig. 1. A total of 14,125 titles/abstracts were screened, 55 full-text articles were reviewed for eligibility, and 26 articles were included in this review [49–74]. Importantly, the 26 articles included data from 22 different studies, because three studies published different outcomes from the same trial in separate articles. The 22 studies that were included in the qualitative synthesis had a total of 713 participants (62% male, 35% female, 3% sex not reported). For study details, see Supplemental material: Table S1 (pages 8-21). In brief, eight studies tested physical education students (mean age across studies weighted by sample size: 22 years; range of mean age in individual studies: 19–24 years), two studies investigated children (10 years; 9–11 years), one study included healthy elderly (mean age 66 years), and other three studies examined adults with type 2 diabetes (53 years; 49–60 years). The remaining eight studies investigated adults (38 years; 21–54 years) with a fitness level of "active" in one and “inactive” in seven studies. Participants received an endurance exercise intervention in eight studies, a strength exercise intervention in eleven studies, and an endurance and strength exercise intervention in three studies. The 191 subjects of the seven studies included in the meta-analysis were homogeneous in terms of sex (98% male, 2% female), background (74% physical education students), and age (mean age across studies weighted by sample size: 25 years; range of mean age in individual studies: 19–33 years). Across all 22 studies included in the systematic review, the chronotype of the participants was reported in 13 studies or 56% of total participants. In these 13 studies, the Horne and Ostberg self-assessment questionnaire was used to assess participants' morningness and eveningness [75]. Of those participants, 9%, 12%, 69%, and 7% were reported to be morning, moderately morning, intermediate, and evening chronotype, respectively. Furthermore, 3% of participants belonged to the moderately morning or intermediate type but were not further specified. Of note, overall, none of the participants were moderately evening chronotype.

### Risk of Bias Within and Across Studies

The risk of bias within studies is displayed in Table 1 [49–52, 54–65, 68–72, 74]. The overall risk of bias observed across all studies was moderate to high. Due to the nature of the study designs, it was not feasible for the study participants and investigators to be both blinded. Further main sources of bias were that only 23% of the studies used the gold standard methods to measure the outcomes. Moreover, only 27%, 9%, and 23% of the studies performed a correction for multiple outcome testing, conducted an appropriate sample size calculation, and registered the study beforehand, respectively. In summary, the main biases in the individual studies are “bias in measurement of the outcome” and “bias in selection of the reported result.” Further, the funnel plot (see Additional file 1: Figure S1—page 22) for those studies included in the meta-analysis indicates that larger and well powered studies are particularly missing. However, due to the limited number of data points (< 10 studies), testing for funnel plot asymmetry cannot be performed and, consequently, no reliable conclusions can be drawn regarding the presence of publication bias [76].

### Results of Individual Studies

There was high heterogeneity in the study populations of the 22 studies included in the systematic review. In detail, children, young physical education students, older adults, and patients with type 2 diabetes (see Additional file 1: Table S1—pages 8–21) were included. In addition, there was also heterogeneity in the type of exercise training. In the endurance training studies, 64%, 18%, 9%, and 9% of the interventions conducted running, cycling, swimming, or a combination of various types of aerobic exercise, respectively. In comparison, in the strength studies, 57%, 7%, and 36% of the interventions focused on the lower body, upper body, and upper and lower body, respectively. The intervention duration (mean duration 9.1 ± 3.7 weeks; range 2–16 weeks), the exercise intensities (see Fig. 2), and exercise frequency (2.7 ± 0.5 sessions per week; range 2–3.5 sessions per week) also varied considerably between studies. In contrast, when considering only the seven studies that were included in the meta-analysis, there was little heterogeneity in the study populations and in the intervention parameters of training intensity (low intensity in 85% of studies) and training frequency (2.8 ± 0.4 sessions per week; range 2–3). For all 22 studies included in the systematic review, the time of day at which morning exercise training occurred was homogeneous, with a mean clock time of 07:58 ± 36 min (range 07:30–09:00), whereas the time of day for afternoon/evening exercise training varied more, with a mean clock time of 17:19 ± 61 min (range 14:30–19:00). The studies differed immensely regarding the outcomes and the methods used to measure them. The anthropometric parameters were measured using a body impedance analyzer, dual-energy X-ray absorptiometry, or a simple digital scale. Aerobic endurance was assessed using VO_2_max with varying protocols and types of exercise (e.g., cycle ergometer versus treadmill ergometer) and distance during a nine-minute running test or six-minute walking test. Anaerobic power or anaerobic capacity was measured using force–velocity test on a cycle ergometer, short swim test as well as 30-s Wingate tests with an equal duration and resistance over the studies. The muscle groups tested in the studies on strength performance were the extensors and flexors of the knee and elbow. Except for one study measuring elbow flexion/extension, all tests to determine the maximum voluntary contraction investigated the knee extensors. However, the methods of measurement differed between isokinetic and isometric dynamometer, as well as resistance machine with a strain gauge. Moreover, maximal and submaximal strength tests (e.g., one-repetition maximum tests or six-repetition maximum tests, respectively) were also commonly performed in the studies. Jump height was measured by a squat jump and a countermovement jump using different measuring devices, i.e., an infrared jump system and/or a vertical jump meter. For the blood analyses, venous and capillary blood was used to determine the main parameters glucose, insulin, HbA1c, testosterone, cortisol, total cholesterol, high-density lipoprotein, low-density lipoprotein, and triglycerides. The comparability of the blood analyses was challenged due to the use of different measurement systems and poor reporting.

### Qualitative Synthesis of Results

Examination of Table 2 reveals that there is no consistent overall finding as well as little statistical evidence for a time-of-day specific influence of training on the listed anthropometric, performance-related, and health-related outcome measures. While no significant results were documented for any anthropometric outcomes, significant differences between morning and evening exercise were reported in four out of ten studies investigating performance-related outcomes. Within the four studies [57, 60, 61, 71], there was no general indication of an advantage for a specific time of day, but rather the results indicated an advantage when time of day of training and testing coincided. In terms of health-related outcomes, two [52, 62] out of seven studies found significant differences between morning and evening exercise. In detail, Brito et al. [52] found a significant decrease in resting systolic blood pressure and Krčmárová et al. [62] reported significant decreases in triglycerides and fasting glucose. Here, evening exercise appeared to be superior to morning exercise.

### Quantitative Synthesis of Results

Figure 3 shows the results of the meta-analysis for the four main categories: strength assessed in the morning (Fig. 3A) and in the evening (Fig. 3B) as well as jump height assessed in the morning (Fig. 3C) and in the evening (Fig. 3D). Only the category jump height assessed in the morning (Fig. 3C) showed a significantly larger overall effect size for time-of-day specific effect of exercise training and provided evidence for a significant superior effect of training in the morning compared to the evening in terms of jump performance improvements in the morning. However, this effect seems to be driven primarily by a single study which is reflected also by the high heterogeneity with *I*^2^ of 80%. No significant differences were found for the remaining three analyses (Fig. 3A, B, D). The exploratory meta-analysis (see Fig. 4) presents the results of comparing congruent testing and training times with incongruent testing and training times for the two outcomes: strength (Fig. 4A) and jump height (Fig. 4B). Both categories show small to medium overall positive effect sizes, indicating possible beneficial improvements when testing and training are congruent, i.e., scheduled at the same time of day. In detail, the overall effect size for strength was 0.22 (95% CI − 0.15 to 0.59). For jump height, the overall effect size was 0.71 (0.00–1.42) and did not include zero, and thus was statistically significant. The comparison of morning exercise and evening exercise shows small and opposite effect sizes. In detail, strength (Fig. 4B) showed a mean difference of 0.13 (95% CI − 0.24 to 0.50), favoring evening exercise, while jump performance (Fig. 4D) showed a mean difference of − 0.38 (95% CI − 0.83 to 0.07), favoring morning exercise. However, the effects were small, non-significant, and mainly driven by a single study.

## Discussion

This systematic review is the first to examine the influence of timing of exercise training on performance-related and health-related outcome measures. The main finding is that there is only little evidence for or against the hypothesis that exercising at a certain time of day is more beneficial than exercising at another time. The qualitative and/or quantitative synthesis of results indicates no superiority of a certain exercise time to improve anthropometrics, cardiovascular health outcomes, cardiometabolic health outcomes, or performance-related outcomes, such as VO_2_max. A major factor is that there are conflicting and inconsistent results between studies, which might be explained by the study heterogeneity and differences in designs. Regarding performance-related outcomes, there is some evidence that performance is positively altered when the timing of testing and training coincide, i.e., morning training improving morning performance more than evening training and evening training improving evening performance more than morning training. However, significant results were only found for the outcome jump height assessed in the morning. Given that the studies included for meta-analysis here only included young male participants, whether such conclusions apply to the general population remains to be examined. Thereby, morning exercise training was significantly superior to evening exercise training in improving morning jump heights in young men. In addition, the exploratory meta-analysis provided supportive statistical evidence that there is an advantage for the outcome jump height when training and testing time coincides.

### Generalizability of the Results and Limitations

From a methodological point of view, this review has several strengths. The review was conducted in accordance with the PRISMA guidelines and was registered beforehand in PROSPERO. An extensive systematic search of relevant databases was performed. The review did not exclude any studies based on publication date, and full texts were made available for all eligible studies. It is the first review on this comprehensive topic that also includes a risk of bias assessment to describe the quality of the individual studies. In addition, a meta-analysis was performed. Although the methodological approach of this review is solid, the generalizability and conclusions drawn from it are limited in some respects. First, the conclusions are not generalizable because in all 22 studies included in the review and in the seven studies examined in the meta-analysis, only 35% and 2% of participants were female, respectively. While cross-sectional studies did not suggest sex differences in diurnal variations in peak performance [77, 78] and one study did not identify a significant interaction between the phase of the menstrual cycle and the time of day on muscle peak strength [79], there are well-known sex differences in physical adaptation to exercise training [31, 80, 81]. Thus, the results of this review need to be interpreted with care and should not be generalized to the female population. Furthermore, the majority of studies investigated young healthy adults and thus cannot be generalized to older or patient populations. In addition, in those studies in which the chronotype was assessed, more than three-quarters of participants belonged to an intermediate chronotype. Participants with an evening chronotype were underrepresented, with 1% of all participants classified as definitely evening chronotype and none as moderately evening chronotype. The low percentage of definitely and moderately evening chronotype in all studies is somewhat unusual, as a previous study of a representative population based on age, socioeconomic status, and gender revealed a Gaussian distribution of chronotype [82]. One possible explanation for the low percentage of evening chronotype in this systematic review is that in some studies only participants with an intermediate chronotype were included. This indicates again that the results need to be interpreted with care and that future studies should include a wider representation of chronotypes, especially evening chronotypes. Furthermore, the questionnaires used in some studies might not be sensitive enough to determine morningness or eveningness in maximum performance [83]. Second, the overall risk of bias in individual studies is rather high, thereby limiting the confidence in some results. The biggest concern is particularly most studies not using the gold standard methodology to assess strength and endurance performance as well as missing correction for testing multiple outcomes leading to “bias in measurement of the outcome” and “bias in selection of the reported result.” Third, the methods that were used and outcomes that were assessed differed widely among studies, making it difficult to perform quantitative synthesis or compare the results from different studies. Fourth, it is unclear to what extent the magnitude of improvements in the respective outcomes is influenced by the type, intensity, and duration of the training intervention itself. The studies differed in the intensity and duration of exercise sessions, the intervention duration, and in the progression of workload over time (see chapter 4.2 for details). Hecksteden et al. [84] provide recommendations on how to report exercise intervention studies; especially, the reporting of crucial information such as frequency, intensity, duration, and type of exercise for each intervention arm, but also precise specifications and documentation on how the intervention was monitored is missing in many studies. As a result of inadequate monitoring of specific intervention factors and large heterogeneity between the exercise interventions, comparisons between studies included in this systematic review are limited.

### Comparability of Interventions

Considering the type of exercise, there was substantial heterogeneity, especially with those studies implementing endurance exercise that were using running, cycling, or swimming. In terms of strength studies, there was more homogeneity with about two thirds of the studies focusing on lower body exercises. Besides the type of exercise, the duration of each exercise session, the exercise intensity, and the total number of exercise sessions (i.e., intervention duration x sessions per week) differed between studies. These three factors can be summarized as training load. While some studies used the first ventilatory threshold to set the exercise intensity [49], others used the respiratory compensation point [52], the rating of perceived exertion [54], or a percentage of VO_2_peak [72] or heart rate reserve [56], while some studies did not report the intensity at all [50, 60, 69]. Thus, drawing conclusions regarding the influence of training loads or performing a dose–response analysis is not possible.

For strength exercise studies, the relevant training characteristics that need to be considered to calculate external load are intensity (e.g., % of maximum or load in kg), frequency (i.e., sessions per week), number of exercises per training session, sets per training session, and repetitions per set [84]. Taking these characteristics into account, we classified each study in this systematic review into low, medium, and high training load. When considering the training load in strength studies, it should be noted that some studies [57–59, 61, 65, 68, 70, 71] pursued a progressive increase in the workload by increasing repetitions or external load over the intervention period, while other studies [51, 62, 69, 72, 74] maintained a consistent workload over the period. A continuous progression of the training load might lead to a higher stimulus and thus to improved adaptations. As a result, the comparability between studies is limited. Important to note is that none of the studies provided information on the actual intensity during the training sessions and on the adherence to the training sessions. Thus, only the planned/intended but not the actual training load has been reported. To achieve comparability between different exercise interventions, a comprehensive and standardized documentation of the parameter of the exercise sessions is required, see Hecksteden et al. [84] and Lambert and Borresen [85].

### Results of Studies Investigating Health-Related Outcomes

The positive impact of physical activity on cardiometabolic and cardiovascular health [86] as well as health-related quality of life [87] has been demonstrated extensively. However, it is unclear whether the time of day of exercise is important for optimizing health. It has been hypothesized in a recent review that exercise timing could be a promising approach in metabolic diseases, because skeletal muscle clocks are very sensitive to exercise and also involved in the expression of glucoregulatory genes [88]. As shown by this systematic review, to date only few studies have examined this issue. Due to the heterogeneity in outcomes and populations, it is difficult to draw conclusions. The influence of the time of day of exercise on markers of the glycemic metabolism has been investigated in four studies [54, 62, 64, 72], with all examining fasting blood glucose and two additionally measuring the markers HbA1c and insulin [64, 72]. The strong association of an impaired glucose metabolism with a diagnosis of type 2 diabetes [89], a disease that is a leading cause of premature death [90], emphasizes the urgency of improving these markers through optimal planned exercise training. Regarding the impact of the time of day of exercise training on glycemic outcomes, Krčmárová et al. [62] reported that there is a higher benefit in fasting blood glucose if the exercise intervention takes place in the evening rather than in the morning. In addition, although no formal statistical analysis was performed, Brooker et al. [54] also suggested that exercise training in the evening is more beneficial for lowering blood glucose levels compared to exercise training in the morning. In contrast, Teo et al. [72] found no statistical differences in fasting glucose, insulin, or HbA1C when morning and evening exercise training was compared. While the three studies have certain similarities in terms of exercise intervention duration and intensity, they vary with respect to the investigated population as well as the type and frequency of the exercise training. The populations ranged from healthy elderly females [62] to inactive and overweight women and men with [72] and without [54, 72] a diagnosis of type 2 diabetes. In addition, the implemented exercise trainings were carried out two to four times a week and varied in the type of training intervention between either strength [62] or endurance [54] only or both combined [72]. When considering measured health-related outcomes, it is important to note that the outcome markers need to be improved throughout the day to achieve optimal health benefits. To accomplish this, the outcomes must be measured at multiple time points throughout the day at baseline and post-intervention. While the three mentioned studies determined fasting blood glucose by venous [62, 72] and capillary [54] blood sampling only at one time point in the morning after overnight fasting, a further study [64] included in this review investigated 24-h blood glucose concentrations using continuous glucose monitoring. Continuous glucose monitoring indirectly estimates the circulating glucose concentration in the blood by measuring the glucose concentration in the interstitial fluid [91]. Since the glucose levels in the interstitial tissue measured by continuous glucose monitoring do not precisely correlate with the blood glucose levels, a direct comparison of the study results is only possible to a limited extent [92, 93]. The result of this crossover study supports the notion that exercise in the afternoon has a larger beneficial effect on blood glucose levels compared to exercise in the morning. Thus, 24-h blood glucose concentrations in men with type 2 diabetes were more improved after a 2-week high-intensity intermittent exercise intervention performed in the afternoon than in the morning [64]. What should be noted here, however, is that the study has a high risk of bias. An additional study [94] reported that a combination of a high fat diet with a moderate- to high-intensity aerobic exercise intervention over five days decreased morning blood glucose levels in the evening training condition compared with morning training condition by a greater magnitude. However, the 24-h blood glucose concentration determined with a continuous glucose monitor showed no reduction in either training condition. With regard to the latter two studies [64, 94], the intervention period was only 2 weeks and five days, respectively. In this short time period, limited physiological adaptation might have occurred, making it impossible to establish a clear conclusion and interpretation of the underlying factors.

Only two studies included in this systematic review [64, 72] assessed insulin as an outcome. The results from the two studies conducted were substantially divergent. While the study of Teo et al. [72] has shown that physical activity lowers insulin levels, the results of Savikj et al. [64] indicated that physical activity increases insulin levels. In contrast, neither study found a difference in whether morning or evening exercise training can contribute to changes in insulin levels in patients with type 2 diabetes mellitus treated with oral hypoglycemic drugs. Although previous studies have identified that high-intensity interval training and moderate-intensity continuous training induce similar effects on insulin dependent outcomes in patients with [95] and without [96] type 2 diabetes, the results of Savikj et al. [64] do not show a positive effect of high-intensity interval training on insulin levels at all—regardless of the time of day of exercise. The positive effect of exercise on insulin-dependent outcomes has been associated with a reduction in body weight or, more specifically, a reduction in regional adipose tissue depots, particularly visceral fat depots [97]. While the majority of subjects in the study by Teo et al. [72] achieved a reduction in body weight from baseline to post-intervention, the study by Savikj et al. [64] failed to document the effect of the exercise intervention on anthropometrics. Thus, whether the absence of the exercise effect on insulin values can be explained by a non-existent change in body composition is unclear. In contrast with the studies by Teo et al. [72] and Savikj et al. [64], a non-randomized study [98] provides evidence for a more positive effect of late afternoon exercise training versus morning exercise training on peripheral insulin sensitivity and fasting blood glucose in adults diagnosed with or at risk for type 2 diabetes. In conclusion, it can only be stated that, according to the current state of research, no definitive statement can be made regarding the impact of the time of day of exercise on insulin-dependent values.

Further, time-of-day specific influence of exercise on lipoproteins and lipids has been investigated in a few studies. In the development of cardiovascular disease, impaired lipid and lipoprotein profiles contribute to the underlying pathology of atherosclerosis [99]. The positive influence and the potential underlying mechanism of physical activity on the blood lipid profile have been demonstrated [100]. However, three studies [54, 62, 64] in this systematic review examining the effect of physical activity on blood lipids failed to identify a substantial positive change in high-density lipoproteins and low-density lipoproteins from baseline to post-intervention. The overall missing positive effect of physical activity on the lipoprotein profile in the study by Krčmárová et al. [62] is unclear and might possibly be due to the intensity of the strength training. In the study by Savikj et al. [64], the short intervention period of only 2 weeks could explain the absence of any effects on the lipoproteins. Regarding the effect of physical activity at different times of day on the outcome triglycerides, the three studies [54, 62, 64] documented divergent results. While triacyglycerides tended to decrease in both the morning and evening training condition in one study [54], they actually showed a tendency to increase in another study [64]. Furthermore, the study by Krčmárová et al. [62] documented that triglycerides in the morning training condition significantly increased from baseline to post-intervention, whereas values in the evening training condition significantly decreased over this time period. The participants in the two training conditions also already showed baseline differences in triglycerides, with significantly lower values in the morning training condition.

The effect of exercise training at a certain time of day was also investigated for the health outcome blood pressure. The study by Brito et al. [52] reported the antihypertensive effect of a 10-week progressive aerobic exercise training on a cycle ergometer in treated hypertensive men. While both the morning and evening training condition lowered their clinical systolic blood pressure values over the intervention period, the evening training condition showed a significantly improved decrease in both morning and evening systolic blood pressure evaluations. A further study [54] similarly showed a greater decrease in systolic blood pressure values in the evening training condition compared with the morning training condition after a 12-week aerobic exercise intervention in inactive, overweight adults. A possible explanation for the greater reduction in systolic blood pressure in the evening training condition in the study by Brito et al. [52] could be the consumption of antihypertensive drugs. All subjects in this study took medication in the morning and about 50% additionally in the evening. It is known that the antihypertensive effect of aerobic exercise is less pronounced when blood pressure levels are lower [101]. Therefore, the participants exercising in the morning trained under the highest effect of antihypertensive drugs during the intervention period and may have experienced lower adaptations as a consequence. However, to confirm this assumption, a clear documentation of individual blood pressure values prior to each training session is required. An improved antihypertensive effect of aerobic exercise in subjects with higher blood pressure levels may also account for the improved decrease in systolic blood pressure that occurred in the evening training condition in the study by Brooker et al. [54]. In this study, the evening training condition already had a 16 mmHg higher resting systolic blood pressure at baseline and therefore trained at higher values than the morning training condition. However, it should be noted that no statistical calculations were performed in this study, and thus, with respect to baseline differences, the conditions were not tested for differences and the results were not adjusted, making it impossible to draw a conclusion based on the collected data and results. Another reason for an impact of the time of day of exercise training on the reduction in blood pressure could be the post-exercise antihypertensive effect, which varies by the time of day. It was shown that in normotensive physically active men, systolic blood pressure, diastolic blood pressure, and mean arterial blood pressure acutely decreased more after 30 min of endurance training at 70% VO_2_peak in the late afternoon than after the identical exercise training in the early morning [102].

### Results of Studies Investigating Performance-Related Outcomes

A reasonable overall analysis of the studies that have investigated performance-related outcomes is hardly possible due to the measurement of different outcomes and the presence of wide variation in the methods. Thus, there is no clear evidence either for or against the hypothesis that exercise timing affects improvements in physical performance. In several studies that examined the same outcome, there was general agreement that 30-s Wingate mean power output improves most when testing and training are congruent. In detail, out of the four studies that examined the effect of low to moderate intensity strength training on 30-s Wingate mean power output, three stated that beneficial improvement is achieved when testing and training occur congruently. Only one study [51] that investigated the influence of 4 weeks of electrostimulation training in active young adults identified a significantly more positive influence of morning training compared to evening training. Other outcomes, such as VO_2_max or electromyography activation, have been investigated in only two studies each. The two studies [52, 54] investigating the effect of training at a certain time of day on VO_2_max both included individuals with risk factors for cardiovascular diseases and contained similar training interventions. Both studies suggested an improvement in VO_2_max through moderate endurance training, independent of the time of day at which the training or testing was performed. Regarding electromyography activation measurements, one study [66] examined inactive adults performing a 10-week lower body strength training program, and another study [61] examined the effects of a 5-week electrical stimulation training program in physical education students, making comparision of results difficult. The first study suggests that morning training is superior, while the second study indicates that the greatest improvement is achieved when testing and training are congruent.

The studies included in the meta-analysis also do not provide evidence for or against a particular time of day being advantageous over another time of day. Rather, as shown in Fig. 3, they also indicate that there is a positive effect when the time of training and testing coincides. The studies included in the meta-analysis investigating strength all had a moderate risk of bias, and four [58, 59, 70, 74] out of five studies suggested that it is an advantage if training and testing time is congruent. Although the generalization of the results of the four studies is poor, the comparability of the results is given. The subjects examined were all male physical education students with a moderately morning or intermediate chronotype. Moreover, the intervention characteristics were quite comparable. In all studies, low-intensity training was performed at approximately 07:30 in the morning and 17:30 in the evening. All but one study [74] trained lower body strength two to three times per week, with squat, leg extension, and leg press being the primary exercises. The only characteristic in which they differ was the duration of the intervention, which ranged from 6 to 14 weeks. One [65] of the five studies showing no tendency to performance enhancement when training and testing time coincide differed in several aspects of the subject characteristics. It investigated diurnally active healthy, and previously untrained men, with a mean age around 30 years old and thus on average 10 years older than the physical education students. Further, no information regarding chronotype was provided.

The four studies [50, 57–59] in the meta-analysis that examined the outcome jump height provided consistent results, with all reporting a beneficial effect when training and testing time is congruent. However, three of the four studies [57–59] were carried out by the same research group. The results therefore need to be reproduced by other research groups before drawing conclusions. Although the studies were very similar in terms of the exercise intervention and study participants and conducted by the same researchers, they differed greatly in the effect size. In detail, the study showing by far the largest effect was the one using the least accurate method (i.e., jump meter) as compared to the others (i.e., infrared system), indicating the importance of high-quality data and the critical risk of bias in many studies.

In conclusion, there is a presumption based on the studies included in this review that improvement in peak performance is greater when training and testing time are congruent. Particularly in competitive sports, where small values account for the difference between victory and defeat, a targeted alignment of training time with competition time can provide a major advantage. This has consistently been concluded in several other reviews examining diurnal variations in maximum performance [22, 25, 30]. However, it should be considered that competition times within a discipline may vary during the season, among others due to time zone differences in international competitions. Furthermore, even within the same events or tournament times of competition can vary often depending on the stage, such as the qualification or knockout rounds taking place earlier during the day and finals taking place at a later time of day and thus closer to prime time on television.

### Possible Underlying Mechanism

Considering the current state of research, it has already been established that both performance-related and health-related outcomes demonstrate diurnal variations. While various health-related outcomes reach their peak and nadir at different times of day, the performance-related outcomes all tend to peak in the late afternoon or evening. Along with several reviews [18–29], almost all studies included in this meta-analysis indicate a nadir in the morning and an acrophase in the late afternoon and evening for the baseline values of performance-related outcomes [57–59, 65, 70, 74]. With regard to the long-term effect, it is impossible to define conclusively whether physical activity at a certain time of day has a greater benefit on health or performance compared to another time of day. Nevertheless, despite a lack of consistent evidence, many original studies included in this review article concluded in their publications that exercise training in the late afternoon and evening may have an advantage over exercise training in the morning. However, our meta-analyses do not show that this conclusion is generalizable or consistent across studies. The actual mechanisms leading to the differences in adaptations between groups exercising in the morning and evening are currently unclear.

The diurnal variations in health-related outcome markers themselves could be a reason for possible differences in adaptations to exercise. Previous investigations have suggested that in the morning, endogenous plasma glucose concentrations are at their peak and whole-body insulin sensitivity is at its best [103]. Furthermore, blood pressure values vary widely throughout the day, depending mainly on the endogenous circadian system [11], behavioral factors such as mental and physical activities, food consumption, and drinking and smoking habits [104]. Whether exercise training has a more positive effect when physiological levels are higher or lower during the time point of exercise is currently uncertain. In addition, existing endogenous circadian rhythms in the post-exercise recovery rate of systolic blood pressure might also have a possible impact. Indeed, faster recovery rates of systolic blood pressures occur in the late evening than at night and in the morning [105]. In order to find a potential causal relationship, future studies need a precise documentation, for example, of blood glucose or blood pressure values before, during and after each training session.

Endogenous and exogenous components could be further possible underlying mechanisms that might lead to an influence of the time of day on acute performance as well as on exercise-induced adaptations. While possible factors leading to diurnal variations in acute peak performance have already been documented in reviews [10, 22], there is a knowledge gap in terms of documenting the underlying mechanisms that could lead to the time of day of training having a longitudinal effect on performance-related and health-related outcomes. One of the main factors that is expected to contribute to diurnal variation in acute physical performance is core body temperature [10, 19], because it shows strong diurnal patterns throughout the day which match the patterns of diurnal variation in performance with a nadir in the early morning and peak in the later afternoon to earlier evening [10]. Further, body core temperature is linked to an improvement in metabolic responses, an increase in connective tissue extensibility, an increase in action potential conduction velocity, a reduction in muscle viscosity [106, 107], an enhanced rate of carbohydrate over fat utilization [108], and encouragement of actin-myosin crossbridge mechanics within the musculoskeletal unit [108]. However, experimental studies increasing or decreasing core body temperature before performance tests indicate that core body temperature is certainly not the only mechanism [109–111]. In addition to core body temperature, other potential underlying mechanisms might be the muscle clock, central clock influences, rhythms of cardiopulmonary function, autonomic nervous system output on muscles, environmental temperature, sleep homeostasis, or nutritional status. However, according to the current state of research, there are few studies investigating the underlying mechanism [24].

Further, it is important to note that most of the studies have inadequate control of confounding factors like diet and sleep. Both are well-known confounders of cardiometabolic and cardiovascular markers [112]. Altered sleep patterns not coinciding with 24-h light–dark cycles lead to changes in endogenous circadian rhythms, which in turn interact with metabolic regulations [113]. Previously, it has been shown that habitual short-term sleep as well as irregular sleep cycles are associated with circadian misalignments and may lead to an increased risk of hypertension [114, 115] or reduced insulin sensitivity [113]. Moreover, it has already been shown that the timing and composition of ingested food affect the circadian systems [116]. All these observations emphasize the importance of monitoring or controlling sleep behavior and dietary patterns in intervention studies.

Differences in the training load or intensity achieved at different times of day could be another confounder. While peak performance is enhanced in the evening in many individuals, the rating of perceived exertion during physical activity shows large variations at different times of day [117, 118]. This is partly due to differences in chronotype [117]. This variation in subject effort between participants exercising at the same time of day might affect subjects’ motivation and thus also adherence to the exercise interventions. This is especially problematic because the documentation of training was poorly recorded in most studies. Within intervention studies targeting training intensities that approximate individual peak performance, it is important to emphasize that although overall it seems that higher performance is achieved in the evening as compared to the morning, it does not mean that every individual reaches their peak in the late afternoon or early evening. This has been demonstrated in studies revealing individual profiles of performance across the day [119–121]. Thus, although peak performance in most subjects is achieved in the late afternoon, some subjects are not at their peak at that point and therefore not at their supposed ideal exercise time. Furthermore, it seems that the time at which individuals habitually exercise also has an impact on physical performance [78] and this could lead to interindividual differences in the increase in performance during the intervention studies.

### Recommendations for Future Studies Investigating the Exercise Time of Day on Performance-Related and Health-Related Outcomes and Perspectives

To generate solid data regarding the time-of-day specific effect of exercise on performance-related and health-related outcomes, further studies with a rigorous methodological approach are needed. We recommend consideration of the following in future studies: (1) inclusion of different populations such as with regard to sex, age, health status, and chronotype to increase generalization, (2) an a priori calculation of the sample size for the primary outcome and statistical adjustment for testing of multiple outcomes, (3) reporting mean differences and effect sizes including 95% confidence intervals for each outcome, and (4) using the gold standard methods to assess each outcome. More detailed recommendations for assessing the performance and baseline and post-intervention can be found in the review by Knaier et al. [24]. Further, we recommend: (5) monitoring of sleep patterns, dietary habits, and unsupervised physical activity with objective methods during the entire intervention period to control for possible confounders. While monitoring of sleep and physical activity with objective methods (primarily wearables) is common these days, there is ongoing discussion about the feasibility of long-term monitoring of dietary habits. One approach for objective monitoring is the remote food photography method, which has shown good feasibility and compliance with just a slight underestimation of energy intake in a free-living setting [122–125]. Furthermore, continuous interstitial glucose monitoring shows promise in validating reported timing of meals [126]. A rigorous monitoring of these behaviors is essential to address another research question that we think is important. We recommend that future studies should address the interaction between the three pillars of health: nutrition, sleep, and physical activity. Because the main focus of research on the effects of exercise timing has been on performance-related outcomes, to this date, there is a paucity of studies investigating the effects of exercise timing on health-related outcomes. Finally, to provide a more holistic understanding of contributing factors to diurnal variation, additional physiological markers, such as blood parameters, body temperature, or muscle clocks, need to be examined.

## Conclusion

There is little evidence for or against the hypothesis that training at a certain time of day is more beneficial in terms of performance-related and health-related outcomes compared to another time of day. However, there is some evidence that there is a benefit when training and testing occurs at the same time of day for performance-related outcomes. At this time, there is insufficient evidence to expand the current training recommendations containing the factors frequency, intensity, type, and time (duration), with the factor time of day of exercise. Nevertheless, the current data clearly indicate that the time of day is not irrelevant and warrants further investigation with a rigorous methodological approach, a broader study population, and adequate controlling and monitoring of all confounding factors.

## Supplementary Information

**Additional file 1**. Supplemental material.

## Data Availability

No data were collected for this review. The detailed search string to replicate the search is provided in the Additional file 1.
