# Supplementary material for: Best Time of Day for Strength and Endurance Training to Improve Health and Performance? A Systematic Review with Meta-analysis
Source: Sports Med Open. 2023 May 19;9:34. doi: 10.1186/s40798-023-00577-5 (PMC10198889; doi:10.1186/s40798-023-00577-5)
Supplement: Supplementary file 1 — Additional file 1. Supplemental material. [file 40798_2023_577_MOESM1_ESM.pdf]

Supplemental material to article

Best time of day for strength and endurance training to improve health and performance?

A systematic review with meta-analysis

Fabienne Bruggisser, Raphael Knaier, Ralf Roth, Wei Wang, Jingyi Qian, Frank A.J.L. Scheer

MATERIALS AND METHODS

The following search strings were used to search the four different databases EMBASE, PubMed, Cochrane Library and SPORTDiscus. The initial search was performed on May 11, 2021 and an updated search was performed on January 4, 2023.

EMBASE: ('circadian rhythm'/exp OR 'circadian rhythm*':ti,ab OR 'time factor'/exp OR 'time factor':ti,ab OR 'time factors':ti,ab OR 'twenty four hour rhythm':ti,ab OR 'twenty four hour rhythms':ti,ab OR 'nyctohemeral rhythm':ti,ab OR 'nyctohemeral rhythms':ti,ab OR 'nycthemeral rhythm':ti,ab OR 'nycthemeral rhythms':ti,ab OR nychtohemeral:ti,ab OR 'phenotype variance':ti,ab OR 'phenotype variation':ti,ab OR 'circadian behavior':ti,ab OR 'body rhythm':ti,ab OR 'body rhythms':ti,ab OR 'endogenous rhythm':ti,ab OR 'endogenous rhythms':ti,ab OR 'diurnal rhythm':ti,ab OR 'diurnal rhythms':ti,ab OR 'diurnal rhythmicity':ti,ab OR 'diurnal cycle':ti,ab OR 'diurnal fluctuation':ti,ab OR 'diurnal fluctuations':ti,ab OR 'diurnal pattern':ti,ab OR 'chronobiology phenomena':ti,ab OR 'chronobiology phenomenon':ti,ab OR 'chronobiology concept':ti,ab OR periodicities:ti,ab OR bioperiodicity:ti,ab OR bioperiodicities:ti,ab OR rhythmicity:ti,ab OR rhythmicities:ti,ab OR cyclicity:ti,ab OR cyclicities:ti,ab OR 'biological rhythm'/exp OR 'biological rhythms':ti,ab OR 'biological rhythm':ti,ab OR 'biologic rhythm':ti,ab OR biorhythm:ti,ab OR biorhythms:ti,ab OR 'biological clock':ti,ab OR 'biological clocks':ti,ab OR 'biologic clock':ti,ab OR 'biologic clocks':ti,ab OR 'biological oscillator':ti,ab OR 'biological oscillators':ti,ab OR 'biological pacemakers':ti,ab OR 'biological pacemaker':ti,ab OR 'endogenous oscillator':ti,ab OR 'endogenous oscillators':ti,ab OR 'biologic pacemaker':ti,ab OR 'biologic pacemakers':ti,ab OR 'biologic oscillator':ti,ab OR 'biologic oscillators':ti,ab OR 'infradian rhythm':ti,ab OR 'light dark cycle':ti,ab OR 'sleep waking cycle':ti,ab OR 'ultradian rhythm':ti,ab OR 'circadian clock':ti,ab OR 'circadian clocks':ti,ab OR 'circadian cycle':ti,ab OR 'circadian fluctuation':ti,ab OR 'circadian fluctuations':ti,ab OR 'circadian periodicity':ti,ab OR 'circadian timing':ti,ab OR 'day night rhythm':ti,ab OR 'time of day':ti,ab OR 'times of day':ti,ab OR 'time of the day':ti,ab OR 'times of the day':ti,ab OR daytimes:ti,ab OR daytime:ti,ab OR 'diurnal variation':ti,ab OR 'diurnal variations':ti,ab OR 'intra day':ti,ab OR 'inter day':ti,ab OR intraday:ti,ab OR interday:ti,ab OR 'circadian variation':ti,ab OR 'circadian variations':ti,ab OR 'circadian phenotype':ti,ab OR 'circadian phenotypes':ti,ab OR 'morning type*':ti,ab OR 'evening type*':ti,ab OR periodicity/exp OR periodicity:ti,ab OR chronotype:ti,ab OR chronotypes:ti,ab OR 'temporal specificity':ti,ab OR 'exercise timing':ti,ab OR 'exercise time':ti,ab OR 'time of exercise':ti,ab OR 'diurnal exercise':ti,ab OR 'morning exercise':ti,ab OR 'evening exercise':ti,ab OR 'afternoon exercise':ti,ab OR 'night exercise':ti,ab OR 'diurnal timing':ti,ab OR 'morning training':ti,ab OR 'evening training':ti,ab OR 'afternoon training':ti,ab OR 'night training':ti,ab OR 'morningness eveningness':ti,ab) AND (exercise/exp OR exercise:ti,ab OR exercises:ti,ab OR exercising:ti,ab OR 'anaerobic exercise':ti,ab OR 'aquatic exercise':ti,ab OR 'arm exercise':ti,ab OR 'closed kinetic chain exercise':ti,ab OR 'cross training':ti,ab OR 'dynamic exercise':ti,ab OR 'isokinetic exercise':ti,ab OR 'leg exercise':ti,ab OR 'muscle exercise':ti,ab OR 'open kinetic chain exercise':ti,ab OR pilates:ti,ab OR plyometrics:ti,ab OR squatting:ti,ab OR 'static exercise':ti,ab OR 'physical activity'/exp OR 'physical activity':ti,ab OR 'physical activities':ti,ab OR climbing:ti,ab OR fighting:ti,ab OR flying:ti,ab OR jumping:ti,ab OR 'nordic walking':ti,ab OR racewalking:ti,ab OR scratching:ti,ab OR 'physical exercise':ti,ab OR 'physical exercises':ti,ab OR 'isometric exercises':ti,ab OR 'isometric exercise':ti,ab OR 'aerobic exercise':ti,ab OR 'aerobic exercises':ti,ab OR 'exercise training':ti,ab OR 'exercise trainings':ti,ab OR 'morning exercise':ti,ab OR 'morning exercises':ti,ab OR 'afternoon exercise':ti,ab OR 'afternoon exercises':ti,ab OR 'evening exercise':ti,ab OR 'evening exercises':ti,ab OR 'diurnal exercise':ti,ab OR 'diurnal exercises':ti,ab OR 'night exercise':ti,ab OR 'night exercises':ti,ab OR gymnastics:ti,ab OR calisthenics:ti,ab OR endurance/exp OR endurance:ti,ab OR 'physical conditioning':ti,ab OR 'human physical training':ti,ab OR 'circuit-based exercise':ti,ab OR 'circuit based exercise':ti,ab OR 'circuit-based exercises':ti,ab OR 'circuit training':ti,ab OR 'endurance training':ti,ab OR 'high intensity interval training':ti,ab OR 'high-intensity interval trainings':ti,ab OR 'high-intensity training':ti,ab OR 'high intensity

training':ti,ab OR 'high-intensity interval':ti,ab OR 'high intensity interval':ti,ab OR hiit:ti,ab OR 'high-intensity intermittent exercise':ti,ab OR 'high-intensity intermittent exercises':ti,ab OR 'sprint interval training':ti,ab OR 'sprint interval trainings':ti,ab OR 'interval training':ti,ab OR 'interval exercise':ti,ab OR 'plyometric exercise':ti,ab OR 'plyometric exercises':ti,ab OR 'plyometric drill':ti,ab OR 'plyometric drills':ti,ab OR 'plyometric training':ti,ab OR 'plyometric trainings':ti,ab OR 'stretch-shortening exercise':ti,ab OR 'stretch shortening exercise':ti,ab OR 'stretch-shortening exercises':ti,ab OR 'stretch-shortening cycle exercise':ti,ab OR 'stretch shortening cycle exercise':ti,ab OR 'stretch-shortening cycle exercises':ti,ab OR 'stretch-shortening drill':ti,ab OR 'stretch shortening drill':ti,ab OR 'stretch-shortening drills':ti,ab OR 'resistance training':ti,ab OR 'strength training':ti,ab OR training:ti,ab OR 'weight lifting'/exp OR 'weight lifting':ti,ab OR 'weight liftings':ti,ab OR 'weight bearing':ti,ab OR 'weight training':ti,ab OR running/exp OR running:ti,ab OR jogging:ti,ab OR 'marathon running':ti,ab OR swimming/exp OR swimming:ti,ab OR walking/exp OR walking:ti,ab OR cycling/exp OR cycling:ti,ab OR intervention:ti,ab OR interventions:ti,ab OR 'exercise intervention':ti,ab OR 'exercise interventions':ti,ab OR 'running intervention':ti,ab OR 'running interventions':ti,ab OR 'walking intervention':ti,ab OR 'walking interventions':ti,ab OR 'walk intervention':ti,ab OR 'walk interventions':ti,ab OR 'sport intervention':ti,ab OR 'sports intervention':ti,ab OR 'continuous training':ti,ab OR 'continuous exercise':ti,ab OR 'continuous endurance training':ti,ab OR 'continuous endurance exercise':ti,ab OR 'agility training':ti,ab OR 'flexibility training':ti,ab OR 'speed training':ti,ab OR fitness:ti,ab OR 'leisure activities':ti,ab OR 'behavior change technique':ti,ab OR 'behavior change techniques':ti,ab OR 'behaviour change technique':ti,ab OR 'behaviour change techniques':ti,ab OR workout:ti,ab OR aerobics:ti,ab OR 'aerobic training':ti,ab) AND ('crossover procedure':de OR 'double-blind procedure':de OR 'randomized controlled trial':de OR 'single-blind procedure':de OR (random* OR factorial* OR crossover* OR cross NEXT/1 over* OR placebo* OR doubl* NEAR/1 blind* OR singl* NEAR/1 blind* OR assign* OR allocat* OR volunteer*):de,ab,ti) NOT (animals/exp NOT humans/exp) NOT [conference abstract]/lim

PubMed: (circadian rhythm[MeSH] OR circadian rhythm*[tiab] OR time factors[tiab] OR time factor[tiab] OR twenty four hour rhythm[tiab] OR twenty four hour rhythms[tiab] OR nyctohemeral rhythm[tiab] OR nyctohemeral rhythms[tiab] OR nycthemeral rhythm[tiab] OR nycthemeral rhythms[tiab] OR nychtohemeral[tiab] OR phenotype variance[tiab] OR phenotype variation[tiab] OR circadian behavior[tiab] OR body rhythm[tiab] OR body rhythms[tiab] OR endogenous rhythm[tiab] OR endogenous rhythms[tiab] OR diurnal rhythm[tiab] OR diurnal rhythms[tiab] OR diurnal rhythmicity[tiab] OR diurnal cycle[tiab] OR diurnal fluctuation[tiab] OR diurnal fluctuations[tiab] OR diurnal pattern[tiab] OR chronobiology phenomena[MeSH] OR chronobiology phenomena[tiab] OR chronobiology phenomenon[tiab] OR chronobiology concept[tiab] OR periodicity[tiab] OR periodicities[tiab] OR bioperiodicity[tiab] OR bioperiodicities[tiab] OR rhythmicity[tiab] OR rhythmicities[tiab] OR cyclicity[tiab] OR cyclicities[tiab] OR biological rhythms[tiab] OR biological rhythm[tiab] OR biologic rhythm[tiab] OR biorhythm[tiab] OR biorhythms[tiab] OR biological clock[tiab] OR biological clocks[tiab] OR biologic clock[tiab] OR biologic clocks[tiab] OR biological oscillator[tiab] OR biological oscillators[tiab] OR biological pacemakers[tiab] OR biological pacemaker[tiab] OR endogenous oscillator[tiab] OR endogenous oscillators[tiab] OR biologic pacemaker[tiab] OR biologic pacemakers[tiab] OR biologic oscillator[tiab] OR biologic oscillators[tiab] OR circadian clock[tiab] OR circadian clocks[tiab] OR circadian cycle[tiab] OR circadian fluctuation[tiab] OR circadian fluctuations[tiab] OR circadian periodicity[tiab] OR circadian timing[tiab] OR day night rhythm[tiab] OR time-of-day[tiab] OR times-of-day[tiab] OR time-of-the-day[tiab] OR times-of-the-day[tiab] OR daytimes[tiab] OR daytime[tiab] OR diurnal variation[tiab] OR diurnal variations[tiab] OR intra-day[tiab] OR inter-day[tiab] OR intraday[tiab] OR interday[tiab] OR circadian variation[tiab] OR circadian variations[tiab] OR circadian phenotype[tiab] OR circadian phenotypes[tiab] OR morning type*[tiab] OR evening type*[tiab] OR chronotype[tiab] OR chronotypes[tiab] OR temporal specificity[tiab] OR exercise timing[tiab] OR exercise time[tiab] OR time-of-exercise[tiab] OR diurnal exercise[tiab] OR morning exercise[tiab] OR evening exercise[tiab] OR afternoon exercise[tiab] OR night exercise[tiab] OR diurnal timing[tiab] OR morning training[tiab] OR evening training[tiab] OR afternoon training[tiab] OR night training[tiab] OR morningness-eveningness[tiab]) AND (exercise[MeSH] OR exercise[tiab] OR exercises[tiab] OR exercising[tiab] OR physical activity[tiab] OR physical activities[tiab] OR physical exercise[tiab] OR physical exercises[tiab] OR isometric exercises[tiab] OR isometric exercise[tiab] OR aerobic exercise[tiab] OR aerobic exercises[tiab] OR exercise training[tiab] OR exercise trainings[tiab] OR morning exercise[tiab] OR morning exercises[tiab] OR afternoon exercise[tiab] OR afternoon exercises[tiab]

OR evening exercise[tiab] OR evening exercises[tiab] OR diurnal exercise[tiab] OR diurnal exercises[tiab] OR night exercise[tiab] OR night exercises[tiab] OR gymnastics[MeSH] OR gymnastics[tiab] OR calisthenics[tiab] OR physical conditioning, human [MeSH] OR physical conditioning[tiab] OR human physical training[tiab] OR circuit-based exercise[tiab] OR circuit based exercise[tiab] OR circuit-based exercises[tiab] OR circuit training[tiab] OR endurance[tiab] OR endurance training[tiab] OR high intensity interval training[tiab] OR high-intensity interval trainings[tiab] OR high-intensity training[tiab] OR high intensity training[tiab] OR high-intensity interval[tiab] OR high intensity interval[tiab] OR HIIT[tiab] OR high-intensity intermittent exercise[tiab] OR high-intensity intermittent exercises[tiab] OR sprint interval training[tiab] OR sprint interval trainings[tiab] OR interval training[tiab] OR interval exercise[tiab] OR plyometric exercise[tiab] OR plyometric exercises[tiab] OR plyometric drill[tiab] OR plyometric drills[tiab] OR plyometric training[tiab] OR plyometric trainings[tiab] OR stretch-shortening exercise[tiab] OR stretch shortening exercise[tiab] OR stretch-shortening exercises[tiab] OR stretch-shortening cycle exercise[tiab] OR stretch shortening cycle exercise[tiab] OR stretch-shortening cycle exercises[tiab] OR stretch-shortening drill[tiab] OR stretch shortening drill[tiab] OR stretch-shortening drills[tiab] OR resistance training[tiab] OR strength training[tiab] OR training[tiab] OR weight lifting[MeSH] OR weight lifting[tiab] OR weight liftings[tiab] OR weight bearing[tiab] OR weight training[tiab] OR running[MeSH] OR running[tiab] OR jogging[tiab] OR marathon running[tiab] OR swimming[MeSH] OR swimming[tiab] OR walking[MeSH] OR walking[tiab] OR bicycling[MeSH] OR bicycling[tiab] OR cycling[tiab] OR intervention[tiab] OR interventions[tiab] OR exercise intervention[tiab] OR exercise interventions[tiab] OR running intervention[tiab] OR running interventions[tiab] OR walking intervention[tiab] OR walking interventions[tiab] OR walk intervention[tiab] OR walk interventions[tiab] OR sport intervention[tiab] OR sports intervention[tiab] OR continuous training[tiab] OR continuous exercise[tiab] OR continuous endurance training[tiab] OR continuous endurance exercise[tiab] OR agility training[tiab] OR flexibility training[tiab] OR speed training[tiab] OR fitness[tiab] OR leisure activities[tiab] OR behavior change technique[tiab] OR behavior change techniques[tiab] OR behaviour change technique[tiab] OR behaviour change techniques[tiab] OR workout[tiab] OR aerobics[tiab] OR aerobic training[tiab] OR anaerobic exercise[tiab] OR aquatic exercise[tiab] OR arm exercise[tiab] OR closed kinetic chain exercise[tiab] OR cross training[tiab] OR dynamic exercise[tiab] OR isokinetic exercise[tiab] OR leg exercise[tiab] OR muscle exercise[tiab] OR open kinetic chain exercise[tiab] OR pilates[tiab] OR plyometrics[tiab] OR resistance training[tiab] OR squatting[tiab] OR static exercise[tiab] OR climbing[tiab] OR fighting[tiab] OR flying[tiab] OR jumping[tiab] OR nordic walking[tiab] OR racewalking[tiab] OR scratching[tiab]) AND (randomized controlled trial[pt] OR controlled clinical trial[pt] OR randomized[tiab] OR placebo[tiab] OR drug therapy[sh] OR randomly[tiab] OR trial[tiab] OR groups[tiab] NOT (animals [mh] NOT humans [mh])) NOT ("animals"[MeSH Terms] NOT "humans"[MeSH Terms])

Cochrane Library: ([mh "circadian rhythm"] OR "circadian rhythm":ti,ab OR "time factors":ti,ab OR "time factor":ti,ab OR "twenty four hour rhythm":ti,ab OR "twenty four hour rhythms":ti,ab OR "nyctohemeral rhythm":ti,ab OR "nyctohemeral rhythms":ti,ab OR "nycthemeral rhythm":ti,ab OR "nycthemeral rhythms":ti,ab OR nychtohemeral:ti,ab OR "phenotype variance":ti,ab OR "phenotype variation":ti,ab OR "circadian behavior":ti,ab OR "body rhythm":ti,ab OR "body rhythms":ti,ab OR "endogenous rhythm":ti,ab OR "endogenous rhythms":ti,ab OR "diurnal rhythm":ti,ab OR "diurnal rhythms":ti,ab OR "diurnal rhythmicity":ti,ab OR "diurnal cycle":ti,ab OR "diurnal fluctuation":ti,ab OR "diurnal pattern":ti,ab OR [mh "chronobiology phenomena"] OR "chronobiology phenomena":ti,ab OR "chronobiology phenomenon":ti,ab OR "chronobiology concept":ti,ab OR periodicity:ti,ab OR periodicities:ti,ab OR bioperiodicity:ti,ab OR bioperiodicities:ti,ab OR rhythmicity:ti,ab OR rhythmicities:ti,ab OR cyclicity:ti,ab OR cyclicities:ti,ab OR "biological rhythms":ti,ab OR "biological rhythm":ti,ab OR "biologic rhythm":ti,ab OR biorhythm:ti,ab OR biorhythms:ti,ab OR "biological clock":ti,ab OR "biological clocks":ti,ab OR "biologic clock":ti,ab OR "biologic clocks":ti,ab OR "biological oscillator":ti,ab OR "biological oscillators":ti,ab OR "biological pacemakers":ti,ab OR "biological pacemaker":ti,ab OR "endogenous oscillator":ti,ab OR "endogenous oscillators":ti,ab OR "biologic pacemaker":ti,ab OR "biologic pacemakers":ti,ab OR "biologic oscillator":ti,ab OR "biologic oscillators":ti,ab OR "circadian clock":ti,ab OR "circadian clocks":ti,ab OR "circadian cycle":ti,ab OR "circadian fluctuation":ti,ab OR "circadian fluctuations":ti,ab OR "circadian periodicity":ti,ab OR "circadian timing":ti,ab OR "day night rhythm":ti,ab OR time-of-day:ti,ab OR times-of-day:ti,ab OR time-of-the-day:ti,ab OR times-of-the-day:ti,ab OR daytimes:ti,ab OR daytime:ti,ab OR "diurnal variation":ti,ab OR "diurnal variations":ti,ab OR intra-day:ti,ab OR inter-day:ti,ab OR intraday:ti,ab OR interday:ti,ab OR

"circadian variation":ti,ab OR "circadian variations":ti,ab OR "circadian phenotype":ti,ab OR "circadian phenotypes":ti,ab OR "morning type":ti,ab OR "evening type":ti,ab OR chronotype:ti,ab OR chronotypes:ti,ab OR "temporal specificity":ti,ab OR "exercise timing":ti,ab OR "exercise time":ti,ab OR time-of-exercise:ti,ab OR "diurnal exercise":ti,ab OR "morning exercise":ti,ab OR "evening exercise":ti,ab OR "afternoon exercise":ti,ab OR "night exercise":ti,ab OR "diurnal timing":ti,ab OR "morning training":ti,ab OR "evening training":ti,ab OR "afternoon training":ti,ab OR "night training":ti,ab OR morningness-eveningness:ti,ab) AND ([mh exercise] OR exercise:ti,ab OR exercises:ti,ab OR exercising:ti,ab OR "physical activity":ti,ab OR "physical activities":ti,ab OR "physical exercise":ti,ab OR "physical exercises":ti,ab OR "isometric exercises":ti,ab OR "isometric exercise":ti,ab OR "aerobic exercise":ti,ab OR "aerobic exercises":ti,ab OR "exercise training":ti,ab OR "exercise trainings":ti,ab OR "morning exercise":ti,ab OR "morning exercises":ti,ab OR "afternoon exercise":ti,ab OR "afternoon exercises":ti,ab OR "evening exercise":ti,ab OR "evening exercises":ti,ab OR "diurnal exercise":ti,ab OR "diurnal exercises":ti,ab OR "night exercise":ti,ab OR "night exercises":ti,ab OR [mh gymnastics] OR gymnastics:ti,ab OR calisthenics:ti,ab OR [mh "physical conditioning, human"] OR "physical conditioning":ti,ab OR "human physical training":ti,ab OR "circuit-based exercise":ti,ab OR "circuit based exercise":ti,ab OR "circuit-based exercises":ti,ab OR "circuit training":ti,ab OR endurance:ti,ab OR "endurance training":ti,ab OR "high intensity interval training":ti,ab OR "high-intensity interval trainings":ti,ab OR "high-intensity training":ti,ab OR "high intensity training":ti,ab OR "high-intensity interval":ti,ab OR "high intensity interval":ti,ab OR HIIT:ti,ab OR "high-intensity intermittent exercise":ti,ab OR "high-intensity intermittent exercises":ti,ab OR "sprint interval training":ti,ab OR "sprint interval trainings":ti,ab OR "interval training":ti,ab OR "interval exercise":ti,ab OR "plyometric exercise":ti,ab OR "plyometric exercises":ti,ab OR "plyometric drill":ti,ab OR "plyometric drills":ti,ab OR "plyometric training":ti,ab OR "plyometric trainings":ti,ab OR "stretch-shortening exercise":ti,ab OR "stretch shortening exercise":ti,ab OR "stretch-shortening exercises":ti,ab OR "stretch-shortening cycle exercise":ti,ab OR "stretch shortening cycle exercise":ti,ab OR "stretch-shortening cycle exercises":ti,ab OR "stretch-shortening drill":ti,ab OR "stretch shortening drill":ti,ab OR "stretch-shortening drills":ti,ab OR "resistance training":ti,ab OR "strength training":ti,ab OR training:ti,ab OR [mh "weight lifting"] OR "weight lifting":ti,ab OR "weight liftings":ti,ab OR "weight bearing":ti,ab OR "weight training":ti,ab OR [mh running] OR running:ti,ab OR jogging:ti,ab OR "marathon running":ti,ab OR [mh swimming] OR swimming:ti,ab OR [mh walking] OR walking:ti,ab OR [mh bicycling] OR bicycling:ti,ab OR intervention:ti,ab OR interventions:ti,ab OR "exercise intervention":ti,ab OR "exercise interventions":ti,ab OR "running intervention":ti,ab OR "running interventions":ti,ab OR "walking intervention":ti,ab OR "walking interventions":ti,ab OR "walk intervention":ti,ab OR "walk interventions":ti,ab OR "sport intervention":ti,ab OR "sports intervention":ti,ab OR "continuous training":ti,ab OR "continuous exercise":ti,ab OR "continuous endurance training":ti,ab OR "continuous endurance exercise":ti,ab OR "agility training":ti,ab OR "flexibility training":ti,ab OR "speed training":ti,ab OR fitness:ti,ab OR "leisure activities":ti,ab OR "behavior change technique":ti,ab OR "behavior change techniques":ti,ab OR "behaviour change technique":ti,ab OR "behaviour change techniques":ti,ab OR workout:ti,ab OR aerobics:ti,ab OR "aerobic training":ti,ab OR "anaerobic exercise":ti,ab OR "aquatic exercise":ti,ab OR "arm exercise":ti,ab OR "closed kinetic chain exercise":ti,ab OR "cross training":ti,ab OR "dynamic exercise":ti,ab OR "isokinetic exercise":ti,ab OR "leg exercise":ti,ab OR "muscle exercise":ti,ab OR "open kinetic chain exercise":ti,ab OR pilates:ti,ab OR plyometrics:ti,ab OR "resistance training":ti,ab OR squatting:ti,ab OR "static exercise":ti,ab OR climbing:ti,ab OR fighting:ti,ab OR flying:ti,ab OR jumping:ti,ab OR "nordic walking":ti,ab OR racewalking:ti,ab OR scratching:ti,ab) NOT ([mh animals] NOT [mh humans])

SPORTDiscus: ((MH "circadian rhythms") OR (TI "circadian rhythm*" OR AB "circadian rhythm*") OR (MH "biological rhythms") OR (TI "biological rhythm*" OR AB "biological rhythm*") OR (TI "circadian behavior" OR AB "circadian behavior") OR (TI "body rhythm" OR AB "body rhythm") OR (TI "body rhythms" OR AB "body rhythms") OR (TI "endogenous rhythm" OR AB "endogenous rhythm") OR (TI "endogenous rhythms" OR AB "endogenous rhythms") OR (TI "time factors" OR AB "time factors") OR (TI "time factor" OR AB "time factor") OR (TI "twenty four hour rhythm" OR AB "twenty four hour rhythm") OR (TI "twenty four hour rhythms" OR AB "twenty four hour rhythms") OR (TI "nyctohemeral rhythm" OR AB "nyctohemeral rhythm") OR (TI "nyctohemeral rhythms" OR AB "nyctohemeral rhythms") OR (TI "nycthemeral rhythm" OR AB "nycthemeral rhythm") OR (TI "nycthemeral rhythms" OR AB "nycthemeral rhythms") OR (TI nychtohemeral OR AB nychtohemeral) OR (TI "phenotype variance" OR AB "phenotype variance") OR (TI "phenotype variation" OR AB "phenotype variation") OR (TI "diurnal rhythm" OR AB

"diurnal rhythm") OR (TI "diurnal rhythms" OR AB "diurnal rhythms") OR (TI "diurnal rhythmicity" OR AB "diurnal rhythmicity") OR (TI "diurnal cycle" OR AB "diurnal cycle") OR (TI "diurnal fluctuation" OR AB "diurnal fluctuation") OR (TI "diurnal pattern" OR AB "diurnal pattern") OR (TI "chronobiology phenomena" OR AB "chronobiology phenomena") OR (TI "chronobiology phenomenon" OR AB "chronobiology phenomenon") OR (TI "chronobiology concept" OR AB "chronobiology concept") OR (TI periodicity OR AB periodicity) OR (TI periodicities OR AB periodicities) OR (TI bioperiodicity OR AB bioperiodicity) OR (TI bioperiodicities OR AB bioperiodicities) OR (TI rhythmicity OR AB rhythmicity) OR (TI rhythmicities OR AB rhythmicities) OR (TI cyclicity OR AB cyclicity) OR (TI cyclicities OR AB cyclicities) OR (TI "biologic rhythm" OR AB "biologic rhythm") OR (TI biorhythm OR AB biorhythm) OR (TI biorhythms OR AB biorhythms) OR (TI "biological clock" OR AB "biological clock") OR (TI "biological clocks" OR AB "biological clocks") OR (TI "biologic clock" OR AB "biologic clock") OR (TI "biologic clocks" OR AB "biologic clocks") OR (TI "biological oscillator" OR AB "biological oscillator") OR (TI "biological oscillators" OR AB "biological oscillators") OR (TI "biological pacemakers" OR AB "biological pacemakers") OR (TI "biological pacemaker" OR AB "biological pacemaker") OR (TI "endogenous oscillator" OR AB "endogenous oscillator") OR (TI "endogenous oscillators" OR AB "endogenous oscillators") OR (TI "biologic pacemaker" OR AB "biologic pacemaker") OR (TI "biologic pacemakers" OR AB "biologic pacemakers") OR (TI "biologic oscillator" OR AB "biologic oscillator") OR (TI "biologic oscillators" OR AB "biologic oscillators") OR (TI "circadian clock" OR AB "circadian clock") OR (TI "circadian clocks" OR AB "circadian clocks") OR (TI "circadian cycle" OR AB "circadian cycle") OR (TI "circadian fluctuation" OR AB "circadian fluctuation") OR (TI "circadian periodicity" OR AB "circadian periodicity") OR (TI "circadian timing" OR AB "circadian timing") OR (TI "day night rhythm" OR AB "day night rhythm") OR (TI time-of-day OR AB time-of-day) OR (TI times-of-day OR AB times-of-day) OR (TI time-of-the-day OR AB time-of-the-day) OR (TI times-of-the-day OR AB times-of-the-day) OR (TI daytimes OR AB daytimes) OR (TI daytime OR AB daytime) OR (TI "diurnal variation" OR AB "diurnal variation") OR (TI "diurnal variations" OR AB "diurnal variations") OR (TI intra-day OR AB intra-day) OR (TI inter-day OR AB inter-day) OR (TI intraday OR AB intraday) OR (TI interday OR AB interday) OR (TI "circadian variation" OR AB "circadian variation") OR (TI "circadian variations" OR AB "circadian variations") OR (TI "circadian phenotype" OR AB "circadian phenotype") OR (TI "circadian phenotypes" OR AB "circadian phenotypes") OR (TI "morning type*" OR AB "morning type*") OR (TI "evening type*" OR AB "evening type*") OR (TI chronotype OR AB chronotype) OR (TI chronotypes OR AB chronotypes) OR (TI "temporal specificity" OR AB "temporal specificity") OR (TI "exercise timing" OR AB "exercise timing") OR (TI "exercise time" OR AB "exercise time") OR (TI time-of-exercise OR AB time-of-exercise) OR (TI "diurnal exercise" OR AB "diurnal exercise") OR (TI "morning exercise" OR AB "morning exercise") OR (TI "evening exercise" OR AB "evening exercise") OR (TI "afternoon exercise" OR AB "afternoon exercise") OR (TI "night exercise" OR AB "night exercise") OR (TI "diurnal timing" OR AB "diurnal timing") OR (TI "morning training" OR AB "morning training") OR (TI "evening training" OR AB "evening training") OR (TI "afternoon training" OR AB "afternoon training") OR (TI "night training" OR AB "night training") OR (TI morningness-eveningness OR AB morningness-eveningness)) AND ((MH exercise) OR (TI exercise OR AB exercise) OR (TI exercises OR AB exercises) OR (TI exercising OR AB exercising) OR (MH "physical activity") OR (TI "physical activity" OR AB "physical activity") OR (TI "physical activities" OR AB "physical activities") OR (TI "physical exercise" OR AB "physical exercise") OR (TI "physical exercises" OR AB "physical exercises") OR (TI "isometric exercises" OR AB "isometric exercises") OR (TI "isometric exercise" OR AB "isometric exercise") OR (TI "aerobic exercise" OR AB "aerobic exercise") OR (TI "aerobic exercises" OR AB "aerobic exercises") OR (TI "exercise training" OR AB "exercise training") OR (TI "exercise trainings" OR AB "exercise trainings") OR (TI "morning exercise" OR AB "morning exercise") OR (TI "morning exercises" OR AB "morning exercises") OR (TI "afternoon exercise" OR AB "afternoon exercise") OR (TI "afternoon exercises" OR AB "afternoon exercises") OR (TI "evening exercise" OR AB "evening exercise") OR (TI "evening exercises" OR AB "evening exercises") OR (TI "diurnal exercise" OR AB "diurnal exercise") OR (TI "diurnal exercises" OR AB "diurnal exercises") OR (TI "night exercise" OR AB "night exercise") OR (TI "night exercises" OR AB "night exercises") OR (MH athletics) OR (TI athletics OR AB athletics) OR (MH gymnastics) OR (TI gymnastics OR AB gymnastics) OR (TI calisthenics OR AB calisthenics) OR (MH "physical training & conditioning") OR (TI "physical training & conditioning" OR AB "physical training & conditioning") OR (TI "human physical training" OR AB "human physical training") OR (TI "circuit-based exercise" OR AB "circuit-based exercise") OR (TI "circuit based exercise" OR AB "circuit based exercise") OR (TI "circuit-based exercises" OR AB "circuit-based exercises") OR (TI "circuit training" OR AB "circuit training") OR (TI endurance OR

AB endurance) OR (TI "endurance training" OR AB "endurance training") OR (TI "high intensity interval training" OR AB "high intensity interval training") OR (TI "high-intensity interval trainings" OR AB "high-intensity interval trainings") OR (TI "high-intensity training" OR AB "high-intensity training") OR (TI "high intensity training" OR AB "high intensity training") OR (TI "high-intensity interval" OR AB "high-intensity interval") OR (TI "high intensity interval" OR AB "high intensity interval") OR (TI HIIT OR AB HIIT) OR (TI "high-intensity intermittent exercise" OR AB "high-intensity intermittent exercise") OR (TI "high-intensity intermittent exercises" OR AB "high-intensity intermittent exercises") OR (TI "sprint interval training" OR AB "sprint interval training") OR (TI "sprint interval trainings" OR AB "sprint interval trainings") OR (TI "interval training" OR AB "interval training") OR (TI "interval exercise" OR AB "interval exercise") OR (TI "plyometric exercise" OR AB "plyometric exercise") OR (TI "plyometric exercises" OR AB "plyometric exercises") OR (TI "plyometric drill" OR AB "plyometric drill") OR (TI "plyometric drills" OR AB "plyometric drills") OR (TI "plyometric training" OR AB "plyometric training") OR (TI "plyometric trainings" OR AB "plyometric trainings") OR (TI "stretch-shortening exercise" OR AB "stretch-shortening exercise") OR (TI "stretch shortening exercise" OR AB "stretch shortening exercise") OR (TI "stretch-shortening exercises" OR AB "stretch-shortening exercises") OR (TI "stretch-shortening cycle exercise" OR AB "stretch-shortening cycle exercise") OR (TI "stretch shortening cycle exercise" OR AB "stretch shortening cycle exercise") OR (TI "stretch-shortening cycle exercises" OR AB "stretch-shortening cycle exercises") OR (TI "stretch-shortening drill" OR AB "stretch-shortening drill") OR (TI "stretch shortening drill" OR AB "stretch shortening drill") OR (TI "stretch-shortening drills" OR AB "stretch-shortening drills") OR (MH "resistance training") OR (TI "resistance training" OR AB "resistance training") OR (TI "strength training" OR AB "strength training") OR (TI training OR AB training) OR (MH "weight lifting") OR (TI "weight lifting" OR AB "weight lifting") OR (TI "weight liftings" OR AB "weight liftings") OR (TI "weight bearing" OR AB "weight bearing") OR (TI "weight training" OR AB "weight training") OR (MH running) OR (TI running OR AB running) OR (TI jogging OR AB jogging) OR (TI "marathon running" OR AB "marathon running") OR (MH swimming) OR (TI swimming OR AB swimming) OR (MH walking) OR (TI walking OR AB walking) OR (MH cycling) OR (TI cycling OR AB cycling) OR (TI intervention OR AB intervention) OR (TI interventions OR AB interventions) OR (TI "exercise intervention" OR AB "exercise intervention") OR (TI "exercise interventions" OR AB "exercise interventions") OR (TI "running intervention" OR AB "running intervention") OR (TI "running interventions" OR AB "running interventions") OR (TI "walking intervention" OR AB "walking intervention") OR (TI "walking interventions" OR AB "walking interventions") OR (TI "walk intervention" OR AB "walk intervention") OR (TI "walk interventions" OR AB "walk interventions") OR (TI "sport intervention" OR AB "sport intervention") OR (TI "sports intervention" OR AB "sports intervention") OR (TI "continuous training" OR AB "continuous training") OR (TI "continuous exercise" OR AB "continuous exercise") OR (TI "continuous endurance training" OR AB "continuous endurance training") OR (TI "continuous endurance exercise" OR AB "continuous endurance exercise") OR (TI "agility training" OR AB "agility training") OR (TI "flexibility training" OR AB "flexibility training") OR (TI "speed training" OR AB "speed training") OR (TI fitness OR AB fitness) OR (TI "leisure activities" OR AB "leisure activities") OR (TI "behavior change technique" OR AB "behavior change technique") OR (TI "behavior change techniques" OR AB "behavior change techniques") OR (TI "behaviour change technique" OR AB "behaviour change technique") OR (TI "behaviour change techniques" OR AB "behaviour change techniques") OR (TI workout OR AB workout) OR (TI aerobics OR AB aerobics) OR (TI "aerobic training" OR AB "aerobic training") OR (TI "anaerobic exercise" OR AB "anaerobic exercise") OR (TI "aquatic exercise" OR AB "aquatic exercise") OR (TI "arm exercise" OR AB "arm exercise") OR (TI "closed kinetic chain exercise" OR AB "closed kinetic chain exercise") OR (TI "cross training" OR AB "cross training") OR (TI "dynamic exercise" OR AB "dynamic exercise") OR (TI "isokinetic exercise" OR AB "isokinetic exercise") OR (TI "leg exercise" OR AB "leg exercise") OR (TI "'muscle exercise" OR AB "muscle exercise") OR (TI "open kinetic chain exercise" OR AB "open kinetic chain exercise") OR (TI pilates OR AB pilates) OR (TI plyometrics OR AB plyometrics) OR (TI "resistance training" OR AB "resistance training") OR (TI squatting OR AB squatting) OR (TI "static exercise" OR AB "static exercise") OR (TI climbing OR AB climbing) OR (TI fighting OR AB fighting) OR (TI flying OR AB flying) OR (TI jumping OR AB jumping) OR (TI "nordic walking" OR AB "nordic walking") OR (TI racewalking OR AB racewalking) OR (TI scratching OR AB scratching)) AND (PT "randomized controlled trial" OR PT "controlled clinical trial" OR (TI randomized OR AB randomized) OR (TI placebo OR AB placebo) OR MW "drug therapy" OR (TI randomly OR AB randomly) OR (TI trial OR AB trial) OR (TI groups OR AB groups) NOT ((MH animals) NOT (MH humans))) NOT ((MH animals) NOT (MH humans))

Supplemental Table S1: Detailed results of individual studies.

Author [Ref]	Study Design<br>Group: N (Sex) / Age (Years)<br>Participants	Exercise Time<br>Frequency (F), Intensity (I), Type (T), Time (T), Intervention duration	Outcome assessment: Method, Time of day	Outcomes (Unit)	Result<br>Group [Test Time]: Pre / Post	p-value / Effect Size / 95% CI for ME vs. EE
**Endurance exercise interventions**						
Alizadeh et al. [49]	parallel group design<br>ME: 23 (f) / 33.6 ± 6.0<br>EE: 19 (f) / 33.9 ± 6.6<br>physically inactive<br>BMI = 25-29.9 kg/m^2<br>dropout rates:<br>ME: 16%, EE: 30%	ME: 08:00-10:00<br>EE: 14:00-16:00<br>F: 3x/week<br>I: HR at VT<br>T: treadmill running<br>T: 30 min<br>6 weeks	body impedance analyzer<br><br>test times not reported	BMI (kg/m^2)<br><br>body fat (%)	ME: 27.29 ± 1.42 / 26.69 ± 1.51<br>EE: 27.57 ± 1.28 / 27.47 ± 1.52<br>ME: 35.71 ± 1.74 / 34.60 ± 1.97<br>EE: 35.71 ± 2.12 / 35.12 ± 2.28	group x week interaction (ME = reference):<br>Estimate (95% CI): 0.50 (0.12, 0.87), p = **0.01**<br>Estimate (95% CI): 0.52 (-0.11, 1.15), p = 0.10
Blonc et al. [50]	parallel group design<br>ME: 7,1 (m,f) / 19.7 ± 1.5<br>EE: 5,3 (m,f) / 20.1 ± 1.3<br>physical education students<br>ME: 10 ± 2 h/week<br>EE: 10.5 ± 2.5 h/week<br>intermediate chronotype	ME: 07:00-09:00<br>EE: 17:00-19:00<br>F: 3x/week<br>I: not reported<br>T: sprints, jumps, track & field exercises<br>T: 60 min<br>5 weeks	7 s force-velocity test (0.060 kg/kg body mass) [Monark 824E, cycle ergometer]<br>SJ and CMJ [Takei Kiki Kogyo vertical jump meter]<br><br>test times all groups: 7:00-09:00, 17:00-19:00	maximal power (W)<br><br>SJ: jump height (cm)<br><br>CMJ: jump height (cm)	ME [07:00]: 781 ± 179 / 857 ± 164<br>EE [07:00]: 826 ± 181 / 823 ± 191<br>ME [17:00]: 809 ± 148 / 854 ± 128<br>EE [17:00]: 801 ± 220 / 862 ± 202<br>ME [07:00]: 46.4 ± 6.2 / 49.3 ± 4.9<br>EE [07:00]: 44.5 ± 1.6 / 43.7 ± 3.4<br>ME [17:00]: 46.7 ± 5.7 / 48.9 ± 7.2<br>EE [17:00]: 44.3 ± 3.4 / 46.7 ± 3.4<br>ME [07:00]: 48.0 ± 8.4 / 49.4 ± 4.9<br>EE [07:00]: 43.4 ± 3.2 / 45.7 ± 3.5<br>ME [17:00]: 48.6 ± 7.9 / 51.3 ± 8.8<br>EE [17:00]: 44.2 ± 4.0 / 46.9 ± 3.3	"…no significant interaction effects of group, time of day, or training for any of the parameters."
Brito et al. [52,53]	parallel group design<br>ME: 15 (m) / 51 ± 8<br>EE: 15 (m) / 49 ± 8<br>Con: 20 (m) / 50 ± 9<br>hypertensive (on medication for at least 4 months, SBP < 160 mmHg, DBP < 105 mmHg), BMI < 35 kg/m^2<br>intermediate chronotype<br>dropout rates:	ME: 07:00-09:00<br>EE: 18:00-20:00<br>F: 3x/week<br>I: HR at anaerobic threshold with increase every 2 weeks to HR 10% below RCP<br>T: cycle ergometer<br>T: increased from 30 to 45 min in the first 4 weeks<br>10 weeks<br>Con: maintenance of the previous level of	incremental test (15 W/min) [cycle ergometer]<br><br>CO_2 rebreathing [metabolic cart, CPX Ultima]<br><br>auscultatory BP	VO_2peak (mL/kg/min)<br><br>cardiac output (L/min)<br><br>resting SBP (mmHg)	ME [07:00]: 21.4 ± 3.2 / 23.1 ± 3.4<br>EE [07:00]: 21.4 ± 3.4 / 23.0 ± 4.6<br>Con [07:00]: 21.1 ± 4.3 / 21.0 ± 4.0<br>ME [20:00]: 22.2 ± 3.2 / 24.5 ± 3.9<br>EE [20:00]: 21.0 ± 4.1 / 23.3 ± 3.8<br>Con [20:00]: 21.7 ± 4.3 / 21.7 ± 2.9<br>ME [07:00]: 4.4 ± 0.7 / 4.4 ± 0.6<br>EE [07:00]: 4.6 ± 0.6 / 4.7 ± 0.5<br>Con [07:00]: 4.6 ± 0.8 / 4.5 ± 0.8<br>ME [18:00]: 4.7 ± 1.1 / 4.8 ± 1.0<br>EE [18:00]: 4.8 ± 1.1 / 4.9 ± 1.0<br>Con [18:00]: 4.7 ± 0.8 / 4.6 ± 0.9<br>ME [07:00]: 133 ± 14 / 128 ± 13<br>EE [07:00]: 129 ± 10 / 123 ± 5	"VO_2peak increased significant and similarly after the MT and ET at both morning and evening tests…"<br><br>[07:00]: p(group) = 0.59, p(phase)= 0.57, p(interaction) = 0.79<br><br>[18:00]: p(group) = 0.67, p(phase) = 0.57, p(interaction) = 0.51<br><br>[07:00]: p(group) = 0.52, p(phase) < **0.001**, p (group vs. phase)= **0.05**; ES: EE vs. ME= -0.45

	ME: 17%, EE: 17%, Con: 0%	activity + 30 min stretching exercise			Con [07:00]: 130 ± 12 / 130 ± 10<br>ME [18:00]: 134 ± 14 / 134 ± 14<br>EE [18:00]: 134 ± 11 / 125 ± 7<br>Con [18:00]: 130 ± 12 / 132 ± 12	[18:00]: p(group) = 0.66, p(phase) = 0.06, p (group vs. phase)< **0.001**; ES:EE vs. ME=-0.61
				resting DBP (mmHg)	ME [07:00]: 90 ± 6 / 90 ± 6<br>EE [07:00]: 91 ± 5 / 87 ± 5<br>Con [07:00]: 88 ± 7 / 89 ± 6<br>ME [18:00]: 91 ± 6 / 91 ± 7<br>EE [18:00]: 92 ± 7 / 89 ± 7<br>Con [18:00]: 90 ± 6 / 91 ± 6	[07:00]: p(group)= 0.56, p(phase) = 0.11, p (group vs. phase)= **0.01**; ES: EE vs. ME=-0.54, [18:00]: p(group)= 0.84, p (phase) = 0.34, p (group vs. phase)= **0.01**; ES: EE vs. ME= -0.33
			HR monitoring [Polar 800cx]<br><br>test times all groups: VO_2max: 7:00-09:00, 20:00-22:00<br>BP and HR recovery: 7:00-09:00, 18:00-20:00	HR recovery at 60 s of recovery (bpm)<br><br>HR recovery at 300 s of recovery (bpm)	data for HR recovery available in graphics only	[07:00]: p(group) = 0.32, p(time) < **0.01,** p(group x time) = **0.05**<br>[18:00]: p(group) = 0.76, p(time) < **0.01,** p(group x time) = 0.20<br>[07:00]: p(group) = 0.13, p(time)= 0.10, p(group x time) = **0.04**<br>[18:00]: p(group) = 0.55, p(time) < **0.01,** p(group x time) < **0.01**
Brooker et al. [54]	parallel group design<br>ME: 2,7 (m,f) / 39 ± 13<br>EE: 4,3 (m,f) / 37 ± 9<br>Con: 4 (f) / 44 ± 11<br>overweight, obese, BMI ≥ 25 kg/m², inactive (activity < 150 min/week)<br>chronotype :<br>ME: morning, intermediate, evening type = 2, 6, 1<br>EE: morning, intermediate, evening type = 3, 3, 1<br>Con: morning, evening type = 3, 1<br>dropout rates:<br>ME: 11%, EE: 0%, Con: 25%	ME: 06:00-09:00<br>EE: 16:00-19:00<br>F: week 1-4: 5x/week, week 5-12: minus 1x week every two weeks until 2x/week was reached<br>I: moderate to vigorous<br>T: self-paced brisk-walking/running on a motor-driven treadmill and additionally not prescribed physical activity (unsupervised sessions)<br>T: minimum of 250 min/week<br>12 weeks<br>Con: maintenance of the previous level of activity	dualenergy x-ray absorptiometry [Hologic Discovery W]<br>indirect calorimetry [TrueOne 2400 Metabolic cart]<br>step-incremental test (Bruce protocol) [treadmill]<br>blood analysis (capillary blood sample)	body fat (%)<br><br>resting metabolic rate (kcal/day)<br><br>VO_2peak (mL/kg/min)<br><br>blood glucose (mmol/L)<br><br>total cholesterol (mmol/L)<br><br>HDL (mmol/L)<br><br>LDL (mmol/L)<br><br>TC:HDL (mmol/L)	ME: 41.7 ± 8.1 / 40.5 ± 9.8<br>EE: 40.6 ± 8.9 / 40.0 ± 8.3<br>Con: 39.7 ± 8.1 / 43.3 ± 7.0<br>ME: 1543 ± 183 / 1520 ± 102<br>EE: 1607 ± 148 / 1550 ± 176<br>Con: 1350 ± 160 / 1305 ± 84<br>ME: 30.7 ± 5.1 / 35.9 ± 8.3<br>EE: 28.5 ± 7.0 / 33.1 ± 8.7<br>Con: 27.5 ± 3.8 / 26.8 ± 4.8<br>ME: 5.6 ± 0.7 / 5.5 ± 0.5<br>EE: 6.0 ± 1.0 / 5.3 ± 0.4<br>Con: 5.1 ± 0.3 / 5.5 ± 0.7<br>ME: 5.4 ± 0.6 / 4.7 ± 0.6<br>EE: 4.7 ± 0.6 / 4.2 ± 0.5<br>Con: 5.2 ± 1.4 / 5.0 ± 0.9<br>ME: 1.7 ± 0.4 / 1.5 ± 0.4<br>EE: 1.0 ± 0.1 / 0.9 ± 0.1<br>Con: 1.6 ± 0.3 / 1.5 ± 0.3<br>ME: 3.1 ± 0.7 / 2.7 ± 0.6<br>EE: 2.6 ± 0.6 / 2.4 ± 0.4<br>Con: 2.7 ± 0.9 / 2.8 ± 0.8<br>ME: 3.3 ± 0.8 / 3.4 ± 0.9<br>EE: 4.3 ± 0.3 / 4.4 ± 0.6<br>Con: 3.3 ± 0.7 / 3.3 ± 0.6	no statistical analyses performed

				triglycerides (mmol/L)	ME: 1.3 ± 0.8 / 1.0 ± 0.4<br>EE: 1.4 ± 1.1 / 1.2 ± 0.5<br>Con: 2.0 ± 1.0 / 1.4 ± 0.2	
			resting blood pressure [automated sphygmomano-meter]	resting SBP (mmHg)	ME: 119 ± 10 / 114 ± 7<br>EE: 135 ± 6 / 121 ± 8<br>Con: 123 ± 28 / 126 ± 22	
			test times not reported	resting DBP (mmHg)	ME: 84 ± 8 / 80 ± 6<br>EE: 86 ± 9 / 84 ± 10<br>Con: 87 ± 16 / 89 ± 16	
Brooker et al. [55]	parallel group design<br>ME: 11,29 (m,f) / 41 ± 12<br>EE: 10,30 (m,f) / 38 ± 11<br>Con: 3,17 (m,f) / 38 ± 10<br>overweight, obese, BMI ≥ 25 kg/m^2, inactive (activity < 150 min/week)<br>chronotype: morning, intermediate, evening type = 30, 46, 23<br>dropout rates:<br>ME: 20%, EE: 18%, Con: 15%	ME: 06:00-09:00<br>EE: 16:00-19:00<br>F: week 1-4: 5x/week, week 5-12: minus 1x week every two weeks until 2x/week was reached<br>I: moderate to vigorous<br>T: self-paced aerobic (treadmill-based) exercise and additionally not prescribed physical activity (unsupervised sessions)<br>T: minimum of 250 min/week<br>12 weeks<br>Con: maintenance of the previous level of activity	dualenergy x-ray absorptiometry [Hologic Discovery W]	body fat (%)	ME: 41.3 ± 7.4 / 40.6 ± 8.1<br>EE: 42.7 ± 7.3 / 41.6 ± 7.6<br>Con: 41.6 ± 6.1 / 41.1 ± 5.9	ME vs. Con (p = 0.747)<br>EE vs. Con (p = 0.802)<br>ME vs. EE (p = 0.484)
			indirect calorimetry [TrueOne 2400 Metabolic cart]	resting metabolic rate (kJ/day)	ME: 6305 ± 845 / 6257 ± 883<br>EE: 6403 ± 1104 / 6457 ± 1043<br>Con: 6285 ± 992 / 6167 ± 1225	p(group) = 0.900, p(time) = 0.990, p(group x time) = 0.951
				resting metabolic rate (kcal/day)	ME: 1509 /1516<br>EE: 1532 / 1527<br>Con: 1504 / 1500	ME vs. Con (p = 0.835)<br>EE vs. Con (p = 0.722)<br>ME vs. EE (p = 0.856)
			step-incremental test (Bruce protocol) [treadmill]	VO_2peak (mL/kg/min)	ME: 29.1 ± 6.3 / 33.6 ± 8.2<br>EE: 28.0 ± 7.5 / 33.2 ± 8.6<br>Con: 28.6 ± 6.7 / 29.9 ± 6.4	ME vs. Con (p = **0.044**)<br>EE vs. Con (p = **0.047**)<br>ME vs. EE (p = 0.820)
			blood analysis (capillary blood sample)	blood glucose (mmol/L)	ME: 5.5 ± 0.5 / 5.5 ± 0.6<br>EE: 5.7 ± 0.5 / 5.6 ± 0.5<br>Con: 5.4 ± 0.4 / 5.5 ± 0.4	p(group) = 0.679, p(time) = 0.085, p(group x time) = 0.410
				total cholesterol (mmol/L)	ME: 4.9 ± 0.9 / 4.1 ± 1.2<br>EE: 4.6 ± 0.8 / 4.7 ± 1.0<br>Con: 5.1 ± 1.0 / 5.1 ± 0.8	p(group) = 0.205, p(time) = 0.051, p(group x time) = 0.023
				HDL (mmol/L)	ME: 1.4 ± 0.5 / 1.4 ± 0.4<br>EE: 1.3 ± 0.4 / 1.3 ± 0.4<br>Con: 1.4 ± 0.4 / 1.5 ± 0.4	p(group) = 0.167, p(time) = 0.585, p(group x time) = 0.874
				LDL (mmol/L)	ME: 2.9 ± 0.8 / 2.6 ± 0.7<br>EE: 2.7 ± 0.7 / 2.8 ± 0.9<br>Con: 2.9 ± 0.7 / 3.0 ± 0.8	p(group) = 0.381, p(time) = 0.439, p(group x time) = 0.510
				TC:HDL (mmol/L)	ME: 3.8 ± 1.2 / 3.5 ± 1.0<br>EE: 3.7 ± 1.0 / 3.9 ± 1.1<br>Con: 3.8 ± 1.2 / 3.7 ± 1.0	p(group) = 0.902, p(time) = 0.145, p(group x time) = 0.654
				triglycerides (mmol/L)	ME: 1.3 ± 0.7 / 1.3 ± 0.7<br>EE: 1.4 ± 0.6 / 1.4 ± 0.8<br>Con: 1.6 ± 0.7 / 1.5 ± 0.7	p(group) = 0.402, p(time) = 0.465, p(group x time) = 0.907
			resting blood pressure [automated sphygmomano-meter]	resting SBP (mmHg)	ME: 120 ± 16 / 118 ± 15<br>EE: 126 ± 16 / 120 ± 10<br>Con: 120 ± 20 / 118 ± 15	p(group) = 0.526, p(time) = **0.006**, p(group x time) = 0.249

			test times not reported	resting DBP (mmHg)	ME: 85 ± 11 / 81 ± 11<br>EE: 85 ± 11 / 82 ± 9<br>Con: 83 ± 12 / 83 ± 10	p(group) = 0.984, p(time) = **0.001**, p(group x time) = 0.076
Chiang et al. [56]	parallel group design<br>combined ME, AE, and EE: 13,7 (m,f) / 48.5 ± 4.2<br>T2D (with at least one oral antidiabetic medication,<br>no insulin therapy), BMI = 26.2 ± 3.9 kg/m^2<br>dropout rates:<br>ME: 0%, EE: 0%, Con: 0%	ME: 08:00-10:00<br>AE: 14:00-16:00<br>EE: 18:00-20:00<br>F: 3x/week<br>I: 70% HR reserve<br>T: treadmill exercise<br>T: 30 min<br>12 weeks	blood analysis (capillary blood sample)<br>test time: before and after each exercise session	before-exercise blood glucose (mg/dL)<br>post-exercise blood glucose (mg/dL)	no data presented in text or tables and no group averages presented	not reported
Ferchichi et al. [60]	parallel group design<br>ME: 6 / 21.8 ± 1.4<br>EE: 6 / 21.8 ± 1.4<br>Con: 6 / 21.8 ± 1.4<br>sex not reported<br>physical education students: university swimming team<br>chronotype: moderately morning, intermediate type = 3, 15	ME: 07:00-08:00<br>EE: 17:00-18:00<br>F: not reported<br>I: not reported<br>T: not reported<br>T: swimming<br>8 weeks<br>Con: no training	swimming performance test (12.5-m swim at maximal velocity using the front crawl stroke)<br>test times for all groups: 7:00-08:00, 17:00-18:00	performance (s)	ME [07:00]: 9.66 ± 0.49 / 7.37 ± 0.30<br>EE [07:00]: 9.87 ± 0.75 / 8.75 ± 0.21<br>Con [07:00]: 9.44 ± 0.76 / 9.24 ± 0.55<br>ME [17:00]: 7.81 ± 0.75 / 7.43 ± 0.54<br>EE [17:00]: 8.26 ± 0.87 / 7.11 ± 0.14<br>Con [17:00]: 8.01 ± 0.82 / 7.83 ± 0.61	time-of-day: F (1.15) = 85.68, p < **0.001** / training: F (1.15) = 55.99, p < **0.001** / group: F (2.15) = 6.62, p < **0.01** / group x training: F (2.15) = 8.75, p < **0.01**
Savikj et al. [64]	crossover design<br>ME: 11 (m) / 60 ± 2<br>EE: 11 (m) / 60 ± 2<br>T2D (no insulin treatment; dietary, metformin treatment = 2, 9), BMI = 27.5 ± 0.6 kg/m^2	ME: 08:00<br>EE: 16:00<br>F: 3x/week<br>I: HIIT (range 180-350 W)<br>T: 6 x 1 min with 1 min recovery between intervals<br>T: cycling<br>2 weeks	continuous glucose monitor [FreeStyle Libre]<br>blood samples (blood chemistry analyses)<br>test times not reported	glucose (mmol/L)<br>HbA1c (%)<br>insulin (pmol/L)<br>total cholesterol (mmol/L)<br>HDL (mmol/L)<br>LDL (mmol/L)	ME: 7.3 ± 1.0 / 7.7 ± 1.3<br>EE: 7.3 ± 1.0 / 7.5 ± 1.0<br>ME: 6.6 ± 1.3 / 6.3 ± 0.7<br>EE: 6.6 ± 1.3 / 6.4 ± 0.7<br>ME: 56.9 ± 30.1 / 71.4 ± 22.9<br>EE: 56.9 ± 30.1 / 70.4 ± 38.5<br>ME: 4.2 ± 1.3 / 4.4 ± 1.0<br>EE: 4.2 ± 1.3 / 4.2 ± 1.3<br>ME: 1.2 ± 0.3 / 1.3 ± 0.3<br>EE: 1.2 ± 0.3 / 1.2 ± 0.3<br>ME: 2.4 ± 1.3 / 2.4 ± 1.3<br>EE: 2.4 ± 1.3 / 2.3 ± 1.3	no effect sizes reported<br>one way ANOVA: p < 0.05 for PTH and TSH; p < 0.1 for T4<br>Tukey's post hoc test: p < 0.1 for PTH and TSH; p , 0.05 for T4

				triacylglycerol (mmol/L)	ME: 1.2 ± 0.7 / 1.6 ± 1.0<br>EE: 1.2 ± 0.7 / 1.4 ± 0.7	
				PTH (pmol/L)	ME: 3.9 ± 0.7 / 4.4 ± 0.7<br>EE: 3.9 ± 0.7 / 4.6 ± 1.0	
				TSH (mU/L)	ME: 1.4 ± 0.7 / 1.7 ± 0.7<br>EE: 1.4 ± 0.7 / 1.9 ± 0.7	
				T4 (pmol/L)	ME: 16.8 ± 2.0 / 16.1 ± 2.3<br>EE: 16.8 ± 2.0 / 15.8 ± 2.3	
				T3 (pmol/L)	ME: 4.7 ± 0.7 / 4.8 ± 0.7<br>EE: 4.7 ± 0.7 / 4.9 ± 0.3	
**Strength exercise interventions**						
Boussetta et al. [51]	parallel group design<br>ME: 8 (m) / 22.0 ± 0.9<br>EE: 8 (m) / 21.2 ± 1.1<br>Con: 8 (m) / 21.1 ± 1.5<br>healthy, active (no history of lower extremity strength training during 3 months before the study), BMI = 23.2 ± 3.1 kg/m^2<br>intermediate chronotype	ME: 07:00-09:00<br>EE: 17:00-18:00<br>F: 3x/week<br>I: not reported<br>T: electrostimulation training of quadriceps femoris:<br>T: 5 s stimulation, 15 s rest period; total 20 min<br>4 weeks<br>Con: no training	knee extension (1RM strength test)	1RM (kg)	ME [07:00]: 106.4 ± 8.2 / 116.0 ± 8.9<br>EE [07:00]: 110.0 ± 7.8 / 113.3 ± 8.4<br>Con [07:00]: 110.9 ± 5.9 / 106.1 ± 3.7<br>ME [17:00]: 109.5 ± 6.5 / 113.3 ± 11.7<br>EE [17:00]: 115.3 ± 4.6 / 113.6 ± 11.7<br>Con [17:00]: 109.9 ± 6.5 / 97.6 ± 14.0	group: F = 5.48, ES = 56.4, p < **0.05**<br>training: F = 13.33, ES = 64.3, p < **0.01** / time-of-day: F = 4.2, ES = 0.06, p > 0.05 / group x training x time of day: F = 3.04, ES = 0.04; p > 0.05
			30 s Wingate test (resistance 0.087 kg/ kg body mass) [cycle ergometer]<br>test times for all groups: 7:00-09:00, 17:00-19:00	peak power output (W/kg)	ME [07:00]: 9.67 ± 0.11 / 11.6 ± 0.08<br>EE [07:00]: 9.55 ± 0.22 / 9.55 ± 0.13<br>Con [07:00]: 9.26 ± 0.20 / 9.46 ± 0.15<br>ME [17:00]: 11.06 ± 0.18 / 12.01 ± 0.20<br>EE [17:00]: 10.87 ± 0.32 / 10.96 ± 0.21<br>Con [17:00]: 10.51 ± 0.22 / 10.94 ± 0.18	group: F = 52.44, ES = 72.4, p < **0.01** / training: F = 15.45, ES = 62.3, p < **0.01** / time-of-day: F = 101.61, ES = 54.2, p < **0.01** / group x training x time-of-day: F = 0.88, ES = 0.49, p < **0.05**
				mean power output (W/kg)	ME [07:00]: 7.92 ± 0.17 / 9.12 ± 0.24<br>EE [07:00]: 8.51 ± 0.13 / 8.39 ± 0.15<br>Con [07:00]: 7.99 ± 0.19 / 7.97 ± 0.18<br>ME [17:00]: 8.53 ± 0.13 / 9.14 ± 0.20<br>EE [17:00]: 9.14 ± 0.16 / 9.11 ± 0.20<br>Con [17:00]: 8.64 ± 0.11 / 8.79 ± 0.06	group: F = 14.13, ES = 59.1, p < **0.01** / training: F = 6.73, ES = 49.5, p < **0.01** / time-of-day: F = 25.98, ES = 48.6, p < **0.01** / group x training x time-of-day: F = 3.10, ES = 0.05, p > 0.05
				fatigue index (%)	ME [07:00]: 47.16 ± 0.49 / 48.60 ± 0.52<br>EE [07:00]: 46.24 ± 0.49 / 46.03 ± 0.52<br>Con [07:00]: 46.50 ± 0.54 / 47.61 ± 0.53<br>ME [17:00]: 49.14 ± 0.53 / 49.42 ± 0.54<br>EE [17:00]: 47.74 ± 0.48 / 49.41 ± 0.49<br>Con [17:00]: 48.15 ± 0.53 / 48.77 ± 0.53	group: F = 0.38, ES = 0.05, p > 0.05 / training: F = 0.78, ES = 0.02, p > 0.05<br>time-of-day: F = 4.24, ES = 0.04, p > 0.05 / group x training x time-of-day: F = 0.02, ES = 0.01, p > 0.05
Chtourou et al. [57]	parallel group design<br>ME: 10 (m) / 22.9 ± 1.3<br>EE: 10 (m) / 22.9 ± 1.3<br>Con: 10 (m) / 22.9 ± 1.3	ME: 07:00-08:00<br>EE: 17:00-18:00<br>F: 3x/week<br>I: week 1-2: 6 x 6 reps at 60-70% of 1RM,<br>2 min rest between sets / week 3-5: 5-6 x 5 reps	30 s Wingate test (resistance 0.087 kg/ kg body mass) [cycle ergometer]	peak power output (W/kg)	ME [07:00]: 10.36 ± 0.82 / 12.04 ± 0.89<br>EE [07:00]: 9.89 ± 0.97 / 9.94 ± 0.97<br>Con [07:00]: 10.68 ± 1.02 / 10.67 ± 1.05<br>ME [17:00]: 10.98 ± 0.72 / 11.71 ± 0.87<br>EE [17:00]: 10.96 ± 0.92 / 11.57 ± 0.69<br>Con [17:00]: 11.38 ± 1.05 / 11.35 ± 1.02	group: F(2.27) = 1.59, ES = 0.1, p > 0.05 / training: F(1.27) = 107.67, ES = 0.8, p < **0.001** / time-of-day: F(1.27) = 64.33, ES = 0.7 p < **0.001** / groups x training x time-of-day: F(2.27) = 57.87, ES = 0.81, p < **0.001**

	physical education students, healthy, no medication<br>chronotype: moderately morning, intermediate type = 8, 22	at 100% of 1RM, 7 min rest between sets / week 6-8: 3 x 3-5 reps at 110-120% of 1RM, 7-9 min rest between sets<br>T: leg extension, leg curl, squat<br>8 weeks<br>Con: maintenance of the previous level of activity		mean power output (W/kg)	ME [07:00]: 7.93 ± 0.47 / 8.87 ± 0.65<br>EE [07:00]: 7.65 ± 0.58 / 7.70 ± 0.58<br>Con [07:00]: 8.21± 0.91 / 8.22 ± 0.75<br>ME [17:00]: 8.21 ± 0.53 / 8.76 ± 0.61<br>EE [17:00]: 8.07 ± 0.49 / 8.77 ± 0.55<br>Con [17:00]: 8.48 ± 0.84 / 8.60 ± 0.68	group: F(2.27) = 1.23, ES = **0.001** , p > 0.05 / training: F(1.27) = 33.65, ES = 0.42, p < **0.001** / time-of-day: F(1.27) = 55.81, ES = 0.6, p < **0.001** / group x training x time-of-day: F(2.27) = 20.95, ES = 0.43, p < **0.001**
			leg extension, leg curl, squat (1RM tests)	1RM leg extension (kg)	ME [07:00]: 71 ± 9.94 / 87.5 ± 7.91<br>EE [07:00]: 69.5 ± 7.98 / 81.5 ± 4.74<br>Con [07:00]: 69 ± 9.66 / 69.5 ± 9.26<br>ME [17:00]: 73.5 ± 8.51 / 87 ± 8.23<br>EE [17:00]: 72 ± 7.53 / 85 ± 4.71<br>Con [17:00]: 72 ± 9.19/ 72 ± 8.88	group: F(2.27) = 3.56, ES = 0.2, p < **0.05** / traininig: F(1.27) = 160.94, ES = 0.85, p < **0.001** / time-of-day: F(1.27) = 44.03, ES = 0.61 p < **0.001** / groups x training x time-of-day: F(2.27) = 2.33, ES = 0.14, p > 0.05
				1RM leg curl (kg)	ME [07:00]: 70 ± 11.3 / 85.5 ± 8.96<br>EE [07:00]: 68.5 ± 10.01 / 81.5 ± 6.68<br>Con [07:00]: 64 ± 9.36 / 64 ± 6.58<br>ME [17:00]: 73 ± 11.11 / 85 ± 7.45<br>EE [17:00]: 71 ± 8.75| / 85 ± 6.66<br>Con [17:00]: 66.5 ± 10.55 / 67 ± 10.05	group: F(2.27) = 6.42, ES = 0.32, p < **0.01** / training: F(1.27) = 188.4, ES = 0.87, p < **0.001** / time-of-day: F(1.27) = 20.16, ES = 0.42, p < **0.001** / groups x training x time-of-day: F(2.27) = 4.5, ES = 0.25, p < **0.05**
				1RM squat (kg)	ME [07:00]: 74 ± 11.97 / 89.5 ± 9.84<br>EE [07:00]: 68 ± 11.1 / 80.5 ± 9.84<br>Con [07:00]: 67.5 ± 10.34 / 67 ± 9.48<br>ME [17:00]: 76.5 ± 11.06 / 88.5 ± 8.51<br>EE [17:00]: 71 ± 10.48 / 84.5 ± 9.55<br>Con [17:00]: 69 ± 10.21 / 69.5 ± 9.84	group: F(2.27) = 4.79, ES = 0.26, p < **0.05** / training: F(1.27) = 302.17, ES = 0.91, p < **0.001** / time-of-day: F(1.27) = 27.98, ES = 0.5, p < **0.001** / groups x training x time-of-day: F(2.27) = 7.83, ES = 0.36, p < **0.01**
			SJ [Takei Kiki Kogyo vertical jump meter]	SJ: jump height (cm)	ME [07:00]: 37.6 ± 4.8 / 52.9 ± 5.5<br>EE [07:00]: 40.0 ± 3.9 / 41.1 ± 3.8<br>Con [07:00]: 41.5 ± 3.4 / 41.6 ± 3.5<br>ME [17:00]: 44.3 ± 4.4 / 52.1 ± 5.5<br>EE [17:00]: 45.0 ± 4.0 / 56.7 ± 3.9<br>Con [17:00]: 48.0 ± 3.9 / 48.0 ± 4.0	group: F(2.27) = 0.52, ES = 0.04, p > 0.05 / training: F(1.27) = 657.41, ES = 0.98, p < **0.001** / time-of-day: F(1.27) = 1453.18, ES = 0.96, p < **0.001** / group x training x time-of-day: F(2.27) = 311.14, ES = 0.96, p < **0.001**
			CMJ [Takei Kiki Kogyo vertical jump meter]<br>test times for all groups: 7:00-08:00, 17:00-18:00	CMJ: jump height (cm)	ME [07:00]: 44.5 ± 4.6 / 62.7 ± 5.0<br>EE [07:00]: 46.3 ± 4.3 / 47.1 ± 4.1<br>Con [07:00]: 47.7 ± 4.6 / 47.9 ± 4.6<br>ME [17:00]: 51.7 ± 4.6 / 61.5 ± 5.8<br>EE [17:00]: 51.6 ± 5.0 / 64.64 ± 4.0<br>Con [17:00]: 55.1 ± 5.1 / 55.31 ± 5.1	group: F(2.27) = 1.72, ES = 0.11, p > 0.05 / training: F(1.27) = 728.52, ES = 0.97, p < **0.001** / time-of-day: F(1.27) = 1146.69, ES = 0.96, p < **0.001** / group x training x time-of-day: F(2.27) = 534.78, ES = 0.97, p < **0.001**
Chtourou et al. [58]	parallel group design<br>ME: 10 (m) / 22.7 ± 2.31<br>EE: 11 (m) / 22.82 ± 1.66<br>Con: 10 (m) / 23.8 ± 1.87	ME: 07:00-08:00<br>EE: 17:00-18:00<br>F: 2-3x/week<br>I: week 1-12: 3 x 10 reps, 2 min recovery between sets / week 13-14: 3 x, 8 reps, 3 min recovery between sets	30 s Wingate test (resistance 0.087 kg/ kg body mass) [cycle ergometer]	peak power output (W/kg)	ME [07:00]: 10.79 ± 1.25 / 11.34 ± 1.04<br>EE [07:00]: 10.89 ± 1.07 / 11.2 ± 1.36<br>Con [07:00]: 11.06 ± 0.87 / 11.08 ± 0.93<br>ME [17:00]: 11.3 ± 1.23 / 11.63 ± 1.6<br>EE [17:00]: 11.58 ± 1.01 /11.98 ± 1.22<br>Con [17:00]: 11.45 ± 0.92 /11.34 ±0.87	concerning training groups:<br>group: F(1.9) = 0.32, ES = 0.12, p > 0.05 / periods: F(2.18) = 8.61, ES = 0.62, p < **0.01** / time-of-day: F(1.9) = 14.54, ES = 0.67, p < **0.01** / groups x periods x time-of-day: F(2.18) = 0.55, ES = 0.09, p > 0.05
				mean power output (W/kg)	ME [07:00]: 8.21 ± 0.95 / 8.42 ± 0.81<br>EE [07:00]: 8.31 ± 0.74 / 8.5 ± 0.8<br>Con [07:00]: 8.13 ± 0.58 / 8.06 ± 0.78	group: F(1.9) = 0.72, ES = 0.03, p > 0.05 / periods effect: F(2.18) = 5.04, ES = 0.38, p < **0.05** / time-of-day effect: F(1.9) = 17.74, ES =

	physical education students, healthy<br>chronotype: moderately morning, intermediate type = 10, 21	T: squat, leg press, leg extension, leg curl<br>12 weeks training (pre- / post) followed by 2 weeks of tapering (post-tapering values not reported in table)<br>Con: no additional training			ME [17:00]: 8.58 ± 0.76 / 8.58 ± 0.83<br>EE [17:00]: 8.76 ± 0.77 / 9 ± 0.78<br>Con [17:00]: 8.39 ± 0.72 / 8.28 ± 0.75	0.78, p < **0.01** / groups x periods x time-of-day: F(2.18) = 0.36, ES = 0.07, p > 0.05
				fatigue index (%)	ME [07:00]: 47.85 ± 9.79 / 47.16 ± 7.48<br>EE [07:00]: 46.76 ± 7.9 / 48.82 ± 9.59<br>Con [07:00]: 51.22 ± 4.78 / 52.18 ± 8.53<br>ME [17:00]: 48.55 ± 6.87 / 49.63 ± 7.72<br>EE [17:00]: 46.62 ± 9.18 / 49.82 ± 8.82<br>Con [17:00]: 50.97 ± 3.97 / 51.42 ± 7.97	group: F(2.18) = 1.03, p > 0.05 / periods: F(2.18) = 1.82, p > 0.05 /<br>time-of-day: F(1.9) = 1.67, p > 0.05 / groups x periods x time-of-day: F(4.36) = 0.42, p > 0.05
			SJ [infrared jump system]	SJ: jump height (cm)	ME [07:00]: 29.5 ± 5.6 / 34.2 ± 4.6<br>EE [07:00]: 31.0 ± 4.2 / 33.6 ± 4.5<br>Con [07:00]: 27.9 ± 3.9 / 27.7 ± 2.1<br>ME [17:00]: 32.5 ± 6.3 / 35.9 ± 5.3<br>EE [17:00]: 33.5 ± 4.5 / 37.7 ± 7.8<br>Con [17:00]: 30.5 ± 5.5 / 30.2 ± 3.4	group: F(1.9) = 0.07, ES = 0.06, p > 0.05 / periods: F(2.18) = 17.32, ES = 0.69, p < **0.001** / time-of-day: F(1.9) = 10.57, ES = 0.66, p < **0.01** / groups x periods x time-of-day: F(2.18) = 2.89, ES = 0.08, p > 0.05
			CMJ [infrared jump system]	CMJ: jump height (cm)	ME [07:00]: 31.9 ± 5.7 / 34.9 ± 4.1<br>EE [07:00]: 33.2 ± 3.3 / 35.3 ± 5.2<br>Con [07:00]: 29.5 ± 3.1 / 30.0 ± 3.4<br>ME [17:00]: 34.3 ± 6.2 / 36.3 ± 6.5<br>EE [17:00]: 36.0 ± 5.0 / 39.0 ± 4.1<br>Con [17:00]: 31.6 ± 4.3 / 32.0 ± 4.2	group: F(1.9) = 1.07, ES = 0.05, p > 0.05 / periods: F(2.18) = 31.2, ES = 0.8, p < **0.001** / time-of-day: F(1.9) = 59.87, ES = 0.78, p < **0.001** , ES = 0.78 / groups x periods x time-of-day: F(2.18) = 0.58, ES = 0.04, p > 0.05
			knee extension dominant leg at ~120° knee flexion [leg extension machine: strain gauge]<br>test times for all groups: 7:00-08:00, 17:00-18:00	MVC (N)	ME [07:00]: 953 ± 196 / 1144 ± 170<br>EE [07:00]: 929 ± 199 / 1046 ± 246<br>Con [07:00]: 885 ± 211 / 887 ± 201<br>ME [17:00]: 1111 ± 161 / 1191 ± 207<br>EE [17:00]: 1022 ± 262 / 1163 ± 282<br>Con [17:00]: 988 ± 148 / 976 ± 199	group: F(1.9) = 0.1, ES = 0.04, p > 0.05 / periods: F(2.18) = 23.97, ES = 0.84, p < **0.001** / time-of-day: F(1.9) = 31.35, ES = 0.87, p < **0.001** / groups x periods x time-of-day: F(2.18) = 13.31, , ES = 0.53, p < **0.001**
Chtourou et al. [59]	parallel group design<br>ME: 10 (m) / 23.1 ± 1.9<br>EE: 11 (m) / 23.1 ± 1.9<br>Con: 10 (m) / 23.1 ± 1.9<br>physical education students<br>(no resistance training during the 6 months before intervention)<br>chronotype: moderately morning, intermediate type = 10, 21	ME: 07:00-08:00<br>EE: 17:00-18:00<br>F: 2-3x/week<br>I: week 1-12: 3 x 10 reps, 2 min recovery between sets / week 13-14: 3 x, 8 reps, 3 min recovery between sets<br>T: squat, leg press, leg extension, leg curl<br>14 weeks followed by 3 and 5 weeks of detraining (not shown in table)	knee extension dominant leg, MVC at ~120° knee flexion [leg extension machine: strain gauge]	MVC (N)	ME [07:00]: 953 ± 192 / 1219 ± 198<br>EE [07:00]: 931 ± 196 / 1092 ± 214<br>Con [07:00]: 886 ± 214 / 886 ± 196<br>ME [17:00]: 1112 ± 159 / 1233 ± 192<br>EE [17:00]: 1022 ± 252 / 1316 ± 204<br>Con [17:00]: 987 ± 151 / 971 ± 177	ME: period: F = 39.8, ES = 0.8, p < **0.001** / time-of-day effect: F = 14.3, ES = 0.6, p < **0.01** /time-of-day x period: F = 6.4, ES= 0.4, p<**0.01**<br>EE: period effect: F = 25.3, ES = 0.7, p < **0.001** / time-of-day: F = 68.4, ES = 0.9, p < **0.001** / time-of-day x period: F = 4.9, ES= 0.3, p< **0.01**
			SJ [infrared jump system]<br>test times for all groups: 7:00-08:00, 17:00-18:00	SJ: jump height (cm)	ME [07:00]: 29.5 ± 5.5 / 36.3 ± 6.1<br>EE [07:00]: 30.9 ± 4.1 / 34.3 ± 6.6<br>Con [07:00]: 27.7 ± 3.9 / 27.8 ± 2.1<br>ME [17:00]: 32.4 ± 6.3 / 36.3 ± 6.3<br>EE [17:00]: 33.5 ± 4.4 / 39.2 ± 5.4<br>Con [17:00]: 30.5 ± 5.4 / 30.0 ± 3.2	ME: period: F = 19.3, ES = 0.7, p < **0.001** / time-of-day: F = 0.9, ES = 0.1, p > 0.05 / time-of-day x period: F = 2.3, ES = 0.2, p > 0.05<br>EE: period: F = 14.5, ES = 0.6, p < **0.001** / time-of-day: F = 21.6, ES = 0.7, p > 0.001 / time-of-day x period: F = 2.3, ES= 0.2, p >0.05

		Con: maintenance of the previous level of activity				
Gueldich et al. [61]	parallel group design<br>ME: 10 (m) / 21.7 ± 2.3<br>EE: 10 (m) / 22.4 ± 1.7<br>physical education students (no previously experienced with electrostimulation training)<br>chronotype: moderately morning, intermediate type = 9, 11	ME: 07:00-08:00<br>EE: 17:00-18:00<br>F: 3x/week<br>I: progressively increase from range, 0-100mA<br>T: 45 x 5s isometric contractions of knee extensor (15 s rest period between), total 15 min<br>T: electrostimulation training<br>5 weeks	knee extension (d leg: MVIC at 60° flexion angle) [leg extension machine: strain gauge]<br>test times for all groups: 07:00, 17:00	MVIC (N)	ME [07:00]: 805 ± 14.5 / 860 ± 13.3<br>EE [07:00]: 801 ± 11.4 / 853 ± 8.2<br>ME [17:00]: 833 ± 10.6 / 865 ± 13.7<br>EE [17:00]: 833 ± 9.8 / 939 ± 5.5	group: F = 1.08, p > 0.05<br>training: F = 93.02, p < **0.001**<br>time-of-day: F = 277.44, p < **0.001**<br>group x training x time-of-day: F = 40.85, p < **0.001**
Krčmárová et al. [62]	parallel group design<br>ME: 10 (f) / 66 ± 4<br>EE: 10 (f) / 66 ± 4<br>Con: 11 (f) / 66 ± 4<br>healthy elderly, physically independent, no orthopedic and cardiac problems<br>dropout rates:<br>ME: 17%, EE: 17%, Con: 8%	ME: 07:30<br>EE: 18:00<br>F: 2x/week<br>I: 3 x 10-12 reps, 2-3 min recovery between sets, 3 min recovery between exercises<br>T: dumbbell bench press, horizontal leg press, seated row, knee extension, lat pull-down, leg curl, machine chest fly, and seated calf raise<br>12 weeks<br>Con: maintenance of the previous level of activity	leg press, seated-row (6RM tests)	6RM leg press (kg)	ME [07:30]: 10.6 ± 2.9 / 15.0 ± 2.5<br>EE [18:00]: 12.5 ± 3.2 / 14.9 ± 3.0<br>Con [n.s.]: 11.4 ± 2.0 / 11.7 ± 1.7	ME vs. Con: 3.38 (2.04, 4.72)<br>EE vs. Con: 2.02 (0.96, 3.08)<br>ME vs. EE: not reported
				6RM seated row exercises (kg)	ME [07:30]: 5.6 ± 1.3 / 8.1 ± 1.3<br>EE [18:00]: 5.1 ± 1.0 / 7.3 ± 1.4<br>Con [n.s.]: 5.2 ± 1.1 / 5.1 ± 0.9	ME vs. Con: 2.85 (1.64, 4.06)<br>EE vs. Con: 2.36 (1.25, 3.47)<br>ME vs. EE: not reported
			30 s sit to stand test (seat height 45 cm)	chair stand (reps)	ME [07:30]: 19 ± 4 / 24 ± 5<br>EE [18:00]: 17 ± 3 / 22 ± 5<br>Con [n.s.]: 15 ± 3 / 14 ± 3	ME vs. Con: 1.85 (0.84, 2.86)<br>EE vs. Con: 1.50 (0.55, 2.45)<br>ME vs. EE: not reported
			30 s arm curl test (dominant hand: 2.3 kg dumbbell)	biceps curls (reps)	ME [07:30]: 23 ± 3 / 29 ± 2.4<br>EE [18:00]: 19 ± 3 / 26 ± 4<br>Con [n.s.]: 21 ± 4 / 20 ± 3	ME vs. Con: (1.46, 3.84)<br>EE vs. Con: (1.08, 3.22)<br>ME vs. EE: not reported
			timed up-and-go test	timed up and go (s)	ME [07:30]: 4.6 ± 0.7 / 3.8 ± 0.9<br>EE [18:00]: 5.1 ± 1.3 / 4.0 ± 1.1<br>Con [n.s.]: 4.2 ± 0.7 / 4.4 ± 0.7	ME vs. Con: (0.73, 2.75)<br>EE vs. Con: (1.04, 3.16)<br>ME vs. EE: not reported
			body impedance analyzer	body fat (%)	ME [morning]: 41.8 ± 4.9 / 39.5 ± 5.7<br>EE [morning]: 42.2 ± 6.8 / 40.4 ± 7.0<br>Con [morning]: 38.5 ± 5.6 / 38.5 ± 5.6	ME vs. Con: (0.87, 2.93)<br>EE vs. Con: (0.54, 2.46)<br>ME vs. EE: not reported
			blood analysis (venous blood sample) in the morning after overnight fast	LDL (mmol/L)	ME [morning]: 2.6 ± 0.8 / 3.0 ± 0.9<br>EE [morning]: 3.4 ± 1.3 / 3.6 ± 1.4	not significant
				sdLDL (mmol/L)	ME [morning]: 0.4 ± 0.2 / 0.8 ± 0.4<br>EE [morning]: 0.7 ± 0.6 / 0.8 ± 0.5	not significant
			test times:<br>strength and functional capacity tests:	HDL (mmol/L)	ME [morning]: 1.5 ± 0.5 / 1.5 ± 0.4<br>EE [morning]: 1.5 ± 0.4 / 1.7 ± 0.4	not significant

			ME: 07:30, EE: 18:00, Con: not reported	blood glucose (mmol/L)	ME [morning]: 5.8 ± 0.4# / 5.6 ± 0.6<br>EE [morning]: 5.6 ± 0.9# / 5.1 ± 0.5‡	p < 0.05
				triglycerides (mmol/L)	ME [morning]: 1.4 ± 0.4# / 1.6 ± 0.4<br>EE [morning]: 1.7 ± 1.0# / 1.3 ± 0.7‡‡	p < 0.01
				hsCRP (mmol/L)	ME [morning]: 2.0 ± 1.2## / 1.8 ± 1.1<br>EE [morning]: 2.5 ± 1.8## / 2.0 ± 1.4	not significant
				total antioxidant status (mmol/L)	ME [morning]: 1.2 ± 0.8 / 1.4 ± 0.1<br>EE [morning]: 1.3 ± 0.1 / 1.5 ± 0.2	not significant
				FSH (mLU/mL)	ME [morning]: 36.6 ± 3.4 / 35.5 ± 3.4<br>EE [morning]: 36.1 ± 5.3 / 34.9 ± 6.7	not significant
				LH (mLU/mL)	ME [morning]: 16.4 ± 6.8 / 18.0 ± 9.5<br>EE [morning]: 15.6 ± 8.3 / 14.4 ± 6.3	not significant
Sedliak et al. [65]	parallel group design<br>ME: 20 (m) / 32 ± 7<br>EE: 18 (m) / 33 ± 7<br>Con: 11 (m) / 34 ± 8<br>healthy, diurnally active healthy, previously untrained (no history of lower extremity strength training)<br>dropout rates:<br>over all groups: 21% dropout, and additional 13% excluded due to non-compliance or incomplete data. No baseline characteristics were reported for excluded subjects.	preparatory training:<br>ME: 17:00-19:00<br>EE: 17:00-19:00<br>time-of-day specific training:<br>ME: 07:00-09:00<br>EE: 17:00-19:00<br>F: 2-3x/week<br>I: week 1-5: high-load neural protocol = 70-85% 1RM (2-4 reps, 3-8 reps) [36% of total volume] / hypertrophic protocol = 60-70% 1RM (2-3 reps, 8-15 reps) [49% of total volume] / explosive protocol = 40-55% 1RM (2 reps, 7-8 reps) [15% of total volume]<br>week 6-10: high-load neural protocol = 80-100% 1RM (2-4 reps, 1-5 reps) [38% of total volume] / hypertrophic protocol = 60-80% 1RM (3-4 reps, 8-12 reps) [40% of total volume] / explosive protocol = 50-60% 1RM (1-3 reps, 5-8 reps) [22% of total volume]	blood analysis (venous blood sample)	cortisol (nmol/L)	ME [07:00]: 601 ± 122 / 519 ± 129<br>ME [12:00]: 372 ± 137 / 339 ± 119<br>ME [17:00]: 300 ± 153 / 216 ± 122<br>ME [20:30]: 150 ± 115 / 122 ± 74<br>EE [07:00]: 625 ± 139 / 603 ± 150<br>EE [12:00]: 332 ± 95 / 309 ± 88<br>EE [17:00]: 270 ± 102 / 220 ± 101<br>EE [20:30]: 113 ± 57 / 109 ± 58<br>Con [07:00]: 562 ± 119 / 518 ± 122<br>Con [12:00]: 312 ± 89 / 305 ± 97<br>Con [17:00]: 235 ± 111 / 219 ± 98<br>Con [20:30]: 77 ± 29 / 102 ± 47	time effect: F(2.5, 115.1) = 619.84, p < **0.001** (time effect was observed at both pre & post, with each time-point within a test day being significantly different from each other (pairwise comparisons, p < **0.001**)<br>pre-post effect: F(1.0, 46.0) = 6.46, p < **0.05**<br>day x time: F(2.8, 129.3) = 5.58, p < **0.01**<br>time x group (between 07:00 and 12:00): F(2.0, 46.0) = 3.28, p < **0.05**
				testosteron (nmol/L)	ME [07:00]: 20.9 ± 5.9 / 20 ± 5.5<br>ME [12:00]: 17.8 ± 5.5 / 16.3 ± 6.2<br>ME [17:00]: 16.9 ± 5.5 / 16.4 ± 6.9<br>ME [20:30]: 13.2 ± 4.7 / 12.6 ± 6.2<br>EE [07:00]: 19.8 ± 6.1 / 17.3 ± 4.1<br>EE [12:00]: 16.5 ± 3.8 / 15.1 ± 3.9<br>EE [17:00]: 14.9 ± 4.7 / 14.9 ± 3.9<br>EE [20:30]: 12.1 ± 2.9 / 11.6 ± 3.1<br>Con [07:00]: 19.3 ± 5.8 / 19.5 ± 6.6<br>Con [12:00]: 17.5 ± 4.6 / 16.8 ± 5.9<br>Con [17:00]: 16.2 ± 3.9 / 16.0 ± 5.8<br>Con [20:30]: 12.1 ± 3.9 / 14.2 ± 3.9	time effect: F (2.3, 105.2)= 132.85; p < **0.001** (during all testing days with gradual decreases from 07:00 until 20:30 h)<br>day x time (between 12:00 & 17:00): F (1.0, 46.0) = 4.26; p < **0.05**<br>no significant main effects of day, pre-post, or group interactions were observed.
			knee extension (right leg MVIC at 120° knee angle) [Leg Ext/Curl Research: biomonitor ME6000T8]<br>test times in all groups: 7:00-08:00, 12:00-	peak torque (Nm)	ME [07:00]: 261 ± 34 / 287 ± 44<br>ME [12:00]: 268 ± 35 / 301 ± 49<br>ME [17:00]: 266 ± 44 / 296 ± 43<br>ME [20:30]: 264 ± 40 / 286 ± 49<br>EE [07:00]: 257 ± 38 / 303 ± 44<br>EE [12:00]: 264 ± 39 / 309 ± 44<br>EE [17:00]: 271 ± 45 / 313 ± 43<br>EE [20:30]: 273 ± 38 / 307 ± 46	pre-post effect: (F(1.0, 25.0) = 26.58, p < **0.001**<br>time effect: F(1.9, 49.7) = 20.10, p < **0.001**<br>pre-post x day: (F(1.0, 25.0) = 6.07, p < **0.05**;<br>day x time: F(2.4, 60.5) = 5.56, p < **0.05**<br>between-group differences were not significant.

		T: half squats, loaded squat jumps, leg presses, knee extensions 10-week preparatory training + 10-week time-of-day specific training<br>Con: maintenance of the previous level of activity	13:00, 17:00-18:00, 20:30-21:30		Con [07:00]: 241 ± 9 / 256 ± 15<br>Con [12:00]: 234 ± 32 / 275 ± 29<br>Con [17:00]: 231 ± 64 / 270 ± 24<br>Con [20:30]: 233 ± 44 / 268 ± 27	pre-post x day x time x group (between 07:00 and 12:00): F(1.0, 25.0) = 5.14; p < **0.05** without and p < 0.052 with the control group included
Sedliak et al. [66]	parallel group design<br>ME: 14 (m) / 32 ± 7<br>EE: 13 (m) / 33 ± 7<br>Con: 7 (m) / 34 ± 8<br>see Sedliak et al. [65] regarding drop-out rates	see Sedliak et al. [65]	half squat (1RM test)<br>test times for all groups: "random" between 9:00 and 16:00. randomization process not described	1RM half squat (kg)	ME: 181 ± 25*/ 193 ± 29*‡<br>EE: 158 ± 13* / 168 ± 16*<br>Con: 153 ± 24* / 157 ± 21	"The interaction between Time-of-day and Group in the relative values reached P=0.061. The other interactions (Pre-to-Post Group, Time-of-day Pre-to-Post, Time-of-day Pre-to-Post Group) were not significant."
Sedliak et al. [67]	parallel group design<br>ME: 9 (m) / 30.6 ± 6.5<br>EE: 7 (m) / 28.6 ± 5.1<br>Con: 8 (m) / 32.4 ± 8.1<br>see Sedliak et al. [65] regarding drop-out rates	see Sedliak et al. [65]	muscle crosssectional area of quadriceps femoris (Magnetic resonance imaging)<br>test times not reported	change in cross-sectional area from baseline to post-intervention (%)	ME: 2.7 ± 0.7<br>EE: 3.5 ± 0.6<br>Con: 0.2 ± 0.3	ME vs. EE (p = 0.188)
Sedliak et al. [68]	parallel group design<br>ME: 11 (m) / 23 ± 2<br>EE: 7 (m) / 24 ± 4<br>Con: 7 (m) / 24 ± 3<br>healthy, untrained (no history of strength training)<br>intermediate type<br>dropout rates:<br>ME: 8%, EE: 42%, Con: 13%	ME: 07:30-08:30<br>EE: 16:00-17:00<br>F: 2x/week<br>I: week 1-5: 3 x 10 to 15 reps, 40 to 60% 1RM / week 6-11: 4 x 8 to 12 reps, 50 to 80% 1RM<br>T: leg presses, knee flexions, knee extensions<br>11 weeks<br>Con: maintenance of the previous level of activity	isokinetic bilateral leg extension (start: 90º in knee joint; end: 0.08 m before legs were fully extended, pedal speed = 0.2 m/s) [dynamometer]<br>blood analysis (venous blood sample)<br>test times: ME: 07:00-08:30, EE: 16:00-17:00, Con: 11:00-13:00	isometric force (N)<br>cortisol (µg/dL)<br>testosterone (ng/mL)	ME [07:00]: 3457 ± 1171 / 4041 ± 1354<br>EE [16:00]: 3656 ± 1588 / 4212 ± 1469<br>Con [11:00]: 3433 ± 1389 / 3342 ± 1142<br>ME [07:00]: 35.6 ± 8.8 / 30.5 ± 6.1<br>EE [16:00]: 31.3 ± 7.9 / 23.6 ± 8.4<br>Con [11:00]: 30.6 ± 8.1 / 24.4 ± 8.5<br>ME [07:00]: 5.85 ± 2.76 / 5.19 ± 1.91<br>EE [16:00]: 5.08 ± 0.91 / 5.36 ± 0.73<br>Con [11:00]: 5.52 ± 2.01 / 6.49 ± 1.93	"There was no statistically significant difference in maximum force production between the training groups at any time points"<br>ME higher cortisol compared to EE (p = 0.081)<br>"Resting total testosterone concentrations were not statistically different among groups at any time point."
Souissi et al. [70]	parallel group design<br>ME: 7 (m) / 19 ± 1.2<br>EE: 7 (m) / 19 ± 1.2	ME: 07:00-08:00<br>EE: 17:00-18:00<br>F: 2x/week	leg extension (1RM test)<br>30 s Wingate test (resistance 0.087 kg/ kg	1RM (kg)<br>peak anaerobic power (W)	ME [07:00]: 39.8 ± 9.5 / 52.4 ± 7.8<br>EE [17:00]: 39.1 ± 7.8 / 52.7 ± 9.2<br>ME [07:00]: 712 ± 31.7 / 832 ± 95.4<br>EE [07:00]: 712 ± 41.9 / 722 ± 69.4	no difference between the absolute increase in 1RM for the ME & EE (p > 0.05)<br>group: F(1.12) = 1.5, p = 0.24; training: F(1.12) = 18.1, p = **0.001 ;** time-of-day:

	physical education students (no strength training programme for the lower body in the 6 months prior to the study)<br>moderate morning and intermediate type	I: week 1: 6 x 6 reps, 60% 1RM, 2 min rest<br>week 2: 8 x 6 reps, 70% 1RM, 2 min rest<br>week 3: 5 x 6 reps, 80% 1RM, 2-3 min rest<br>week 4: 5 x 3 reps, 90% 1RM, 3-4 min rest<br>week 5: 5 x 3 reps, 93% 1RM, 3-4 min rest<br>week 6: 3 x 3 reps, 95% 1RM, 3-5 min rest<br>T: leg extension<br>6 weeks	body mass) [cycle ergometer]		ME [17:00]: 772 ± 85.6 / 833 ± 116<br>EE [17:00]: 741 ± 49.3 / 809 ± 52.0	F(1.12) = 16.8, p = **0.001;** group x training x time-of-day: F(1.12) = 10.8, p = 0.006
			knee extension (instantaneous muscular torques within the Range of Motion of 0-90°) [isokinetic dynamometer]<br>test times for all groups: knee extension and Wingate test: 7:00-08:00, 17:00-18:00<br>test times for RM: ME: 07:00-08:00, EE: 17:00-18:00	peak torque (N*m): 1.05 rad/s	ME [07:00]: 215 ± 47.4 / 261 ± 40.9<br>EE [07:00]: 200 ± 36.7 / 231 ± 27.8<br>ME [17:00]: 214 ± 43.3 / 256 ± 40.3<br>EE [17:00]: 202 ± 30.7 / 256 ± 35.1	group: F(1.12) = 0.07, p = 0.78; training: F(1.12) = 94.8, p < **0.001 ;** time-of-day: F(1.12) = 33.5, p < **0.001;** velocity: F(5.60) = 479, p < **0.001;** training groups x angular velocities: F(5.60) = 1.3, p = 0.26<br>velocity x training: F(5.60) = 11.1, p < **0.001**<br>time-of-day: F(1.12) = 33.5, p < **0.001**<br>group x training x time-of-day interaction: F(1.12) = 4.76, p = **0.01**
				peak torque (N*m): 2.10 rad/s	ME [07:00]: 168 ± 40.2 / 206 ± 31.9<br>EE [07:00]: 157 ± 34.1 / 193 ± 18.5<br>ME [17:00]: 173 ± 30.1 / 202 ± 31.1<br>EE [17:00]: 172 ± 18.6 / 206 ± 27.7	
				peak torque (N*m): 3.14 rad/s	ME [07:00]: 130 ± 41.2 / 169 ± 22.9<br>EE [07:00]: 136 ± 23.1 / 160 ± 16.7<br>ME [17:00]: 139 ± 17.0 / 173 ± 31.4<br>EE [17:00]: 145 ± 19.4 / 173 ± 22.8	
				peak torque (N*m): 4.19 rad/s	ME [07:00]: 112 ± 39.7 / 146 ± 25.8<br>EE [07:00]: 117 ± 22.3 / 133 ± 13.3<br>ME [17:00]: 121 ± 14.9 / 146 ± 31.2<br>EE [17:00]: 125 ± 28.3 / 146 ± 19.7	
				peak torque (N*m): 5.24 rad/s	ME [07:00]: 91.2 ± 35.5 / 122 ± 27.8<br>EE [07:00]: 94.2 ± 25.4 / 110 ± 14.5<br>ME [17:00]: 104 ± 31.5 / 119 ± 27.7<br>EE [17:00]: 104 ± 13.0 / 123 ± 15.1	
				peak torque (N*m): 6.29 rad/s	ME [07:00]: 78.1 ± 25.9 / 100 ± 23.9<br>EE [07:00]: 80.4 ± 18.1 / 91.0 ± 16.9<br>ME [17:00]: 84.2 ± 27.6 / 100 ± 26.2<br>EE [17:00]: 87.0 ± 18.6 / 103 ± 13.3	
Souissi et al. [71]	parallel group design<br>ME: 8 (m) / 10-11<br>EE: 8 (m) / 10-11<br>Con: 8 (m) / 10-11<br>healthy, untrained<br>dropout rate: 0%	ME: 07:00-08:00<br>EE: 17:00-18:00<br>F: 2x/week<br>I: week 1-2: 2 x 10 reps at 50% 1RM<br>week 3-6: 2 x 10 reps at 60% 1RM<br>T: bench press, hack squat, leg extension, leg curl, pullover machine<br>6 weeks	30 s Wingate test (resistance 0.087 kg/ kg body mass) [cycle ergometer]	peak power output (W/kg)	ME [07:00]: 8.57 ± 0.97 / 9.23 ±0.97<br>EE [07:00]: 7.99 ± 1.3 / 8.26 ± 1.25<br>Con [07:00]: 8.15 ± 1.2 / 8.22 ± 1.01<br>ME [17:00]: 9.11 ± 1.01 / 9.39 ±1.14<br>EE [17:00]: 8.66 ± 1.19 / 9.28 ±1.43<br>Con [17:00]: 8.67 ± 1.15 / 8.74 ± 1.05	group effect: F(2.14) = 0.68, p > 0.05 / training effect: F(1.7) = 38.67, p < **0.001** / time-of-day: F(1.7) = 73.7, p < **0.001** /groups x pre-/post-training x time-of-day: F(2.14) = 3.92, p < **0.05**
				mean power output (W/kg)	ME [07:00]: 7.12 ± 0.64 / 7.63 ± 0.68<br>EE [07:00]: 7.01 ± 0.85 / 7.22 ± 1.00<br>Con [07:00]: 6.89 ± 0.81 / 6.9 ± 0.75<br>ME [17:00]: 7.48 ± 0.59 / 7.61 ± 0.51<br>EE [17:00]: 7.48 ± 0.97 / 7.98 ± 1.06<br>Con [17:00]: 7.24 ± 0.54 / 7.23 ± 0.35	group effect: F(1.7) = 0.81, p > 0.05 / training effect: F(1.7) = 39.71, p < **0.001** / time-of-day effect: F(1.7) = 27.5, p < **0.01** / groups x training x time-of-day: F(2.14) = 3.88, p < **0.05**
				fatigue index (%)	ME [07:00]: 0.34 ± 0.1 / 0.33 ± 0.07<br>EE [07:00]: 0.32 ± 0.07 / 0.34 ± 0.08<br>Con [07:00]: 0.39 ± 0.09 / 0.36 ± 0.09<br>ME [17:00]: 0.35 ± 0.11 / 0.36 ± 0.11<br>EE [17:00]: 0.33 ± 0.04 / 0.35 ± 0.06	group: F(2.14) = 1.51, p > 0.05 / training effect: F(1.7) = 0.1, p > 0.05 / time-of-day: F(1.7) = 2.88, p > 0.05 / groups × training × time-of-day: F(2.14) = 0.06, p > 0.05)

					Con [17:00]: 0.43 ± 0.12 / 0.4 ± 0.11	
			knee extension (dominant leg: MVC at ~120° knee flexion) [leg extension machine: strain gauge]	MVC (N)	ME [07:00]: 325 ± 128 / 486 ± 117<br>EE [07:00]: 352 ± 51 / 407 ± 89<br>Con [07:00]: 331 ± 74 / 336 ± 70<br>ME [17:00]: 395 ± 132/485 ± 152<br>EE [17:00]: 405 ± 82 / 521 ± 84.3<br>Con [17:00]: 386 ± 75 / 385 ± 79	group: F(2.14) = 1.68, p > 0.05 / training: F(1.7) = 30.31, p < **0.001** / time-of-day: F(1.7) = 22.84, p < **0.01** / groups x training x time-of-day: F(2.14) = 3.87, p < **0.05**
				MVC (N/kg)	ME [07:00]: 7.6 ± 1.6 / 11.6 ± 2.1<br>EE [07:00]: 8.4 ± 1.5 / 9.5 ± 1.7<br>Con [07:00]: 7.9 ± 1.7 / 8.0 ± 1.6<br>ME [17:00]: 9.3 ± 1.8 / 11.4 ± 2.2<br>EE [17:00]: 9.7 ± 2.3 /12.3 ± 2.7<br>Con [17:00]: 9.3 ± 1.9 / 9.2 ± 2.2	
			SJ [infrared jump system]	SJ: jump height (cm)	ME [07:00]: 21.5 ± 3.4 / 25.0 ± 3.9<br>EE [07:00]: 19.0 ± 4.2 / 19.5 ± 3.8<br>Con [07:00]: 21.3 ± 4.2 / 20.9 ± 2.6<br>ME [17:00]: 24.9 ± 4.3 / 25.1 ± 3.7<br>EE [17:00]: 21.8 ± 4.1 / 24.2 ± 4.8<br>Con [17:00]: 23.8 ± 4.5 / 24.2 ± 4.3	group: F(2.14) = 2.56, p > 0.05 / training: F(1.7) = 13.52, p < **0.01** / time-of-day: F(1.7) = 44.01, p < .001 / group x training x time-of-day: F(2.14) = 5.49, p < **0.05**
			CMJ [infrared jump system]<br><br>test times for all groups: 07:00, 17:00	CMJ: jump height (cm)	ME [07:00]: 22.4 ± 4.1 / 27.0 ± 4.5<br>EE [07:00]: 19.6 ± 4.3 / 21.5 ± 4.6<br>Con [07:00]: 22.9 ± 4.0 / 22.9 ± 3.6<br>ME [17:00]: 26.5 ± 6.1 / 27.5 ± 3.7<br>EE [17:00]: 23.6 ± 4.4 / 27.2 ± 3.6<br>Con [17:00]: 25.3 ± 5.4 / 25.3 ± 3.9	group: F(2.14) = 1.78, p > 0.05 / training effect: F(1.7) = 13.78, p < **0.01**<br>time-of-day: F(1.7) = 76.11, p < **0.001** / groups x training x time-of-day: F(2.14) = 3.84, p < **0.05**
Zbidi et al. [74]	parallel group design<br>ME: 10 (m) / 23.6 ± 2.6<br>EE: 10 (m) / 23.7 ± 4.8<br>physical education students (no history of upper or lower extremity strength training during the 6 months before the study)<br>chronotype: moderately morning, intermediate type = 6, 14	ME: 07:00-08:00<br>EE: 17:00-18:00<br>F: 3x/week<br>I: maximal isometric voluntary co-contractions<br>T: 6 x 8 reps, 30 s recovery between reps, 2 min recovery between sets<br>T: training of elbow antagonistic muscle pairs (90° angle)<br>6 weeks	elbow flexion and extension (MVF & MRFD: unilateral isometric voluntary contraction of elbow flexor and extensor muscles at 90° elbow angle)<br><br>test times for all groups: 07:00, 17:00	MVF (N): elbow flexion	ME [07:00]: 284 ± 43 / 317 ± 42<br>EE [07:00]: 266 ± 60 / 279 ± 62<br>ME [17:00]: 297 ± 45 / 318 ± 46<br>EE [17:00]: 281 ± 69 / 308 ± 71	group: F(1.18)=0.6, p=0.4/ training: F(1.18) = 137, ES= 0.88, p< **0.001**/ time-of-day: F(1.18) =22.9, ES= 0.56, p< **0.001** / group x training x time-of-day:F(1.18)= 27.8, ES= 0.60, p< **0.001**
				MVF (N): elbow extension	ME [07:00]: 228 ± 43 / 267 ± 48<br>EE [07:00]: 219 ± 39 / 233 ± 42<br>ME [17:00]: 243 ± 53 / 265 ± 55<br>EE [17:00]: 230 ± 36 / 262 ± 47	group: F(1.18)= 0.53, p= 0.4/ training: F(1.18) =155, ES= 0.89, p< **0.001**/ time-of-day:F(1.18) =33.5, ES= 0.65, p< **0.001** / group x training x time-of-day:F(1.18) = 67.2, ES= 0.78, p<**0.001**
				MRFD (N/s): elbow flexion	ME [07:00]: 2964 ± 407 / 3033 ± 458<br>EE [07:00]: 2548 ± 644 / 2581 ± 560<br>ME [17:00]: 3055 ± 382 / 3083 ± 415<br>EE [17:00]: 2775 ± 772 / 2852 ± 678	time-of-day: F(1.18)= 23.2, ES= 0.56, p<**0.001** / group x training x time-of-day: F(1.18)= 4.88, ES = 0.21, p= 0.04 / no effect of group and training (> 0.05)
				MRFD (N/s): elbow extension	ME [07:00]: 2291 ± 323 / 2336 ± 357<br>EE [07:00]: 2165 ± 459 / 2225 ± 400<br>ME [17:00]: 2389 ± 323 / 2408 ± 332<br>EE [17:00]: 2385 ± 536 / 2433 ± 493	time-of-day: F(1.18) = 28.59, ES = 0.61, p < **0.001** / group x time-of-day: F(1.18) = 5.81, ES = 0.24, p = 0.02
**Endurance and Strength exercise interventions**						

Saidi et al. [63]	parallel group design<br>ME: 4, 12 (m,f) / 54.7 ± 8.5<br>EE: 4, 8 (m,f) / 53.5 ± 7.5<br>overweight, obese, BMI ≥ 25 kg/m², inactive (activity < 120 min/week)<br>dropouts:<br>ME: 11%, EE: 33%<br>no baseline characteristics were reported for excluded subjects	ME: 09:00<br>EE: 18:30<br>F: 3x/week<br>I: 60% maximum HR and 60% 1RM (initial)<br>T: 90 min<br>T: combination of aerobic exercise and strengthening exercises for the major muscles<br>12 weeks	body impedance analyzer<br>six-minute walking test<br>30 s wall press test<br>30 s sit to stand test<br>test time for body impedance analyzer: during the morning<br>further test times not reported	body fat (%)<br>estimated VO_2max (mL/min/kg)<br>presses (reps)<br>chair stand (reps)	ME: 42.4 ± 7.8 / 40.7 ± 7.9<br>EE: 42.1 ± 8.0 / 39.6 ± 8.6<br>ME: 25.7 ± 7.1 / 40.6 ± 16.5<br>EE: 24.3 ± 7.1 / 39.0 ± 15.6<br>ME: 18.8 ± 4.6 / 21.7 ± 5.0<br>EE: 19.3 ± 4.3 / 20.2 ± 3.9<br>ME: 17.4 ± 6.8 / 20.3 ± 6.8<br>EE: 21.5 ± 6.4 / 20.4 ± 5.0	within-group difference ($p = 0.003$)<br>within-group difference ($p = 0.04$)<br>between-group difference ($p = 0.35$)<br>within-group difference ($p < 0.001$)<br>within-group difference ($p < 0.001$)<br>between-group difference ($p = 0.95$)<br>within-group difference ($p < 0.01$)<br>within-group difference ($p = 0.30$)<br>between-group difference ($p = 0.23$)<br>within-group difference ($p < 0.02$)<br>within-group difference ($p = 0.57$)<br>between-group difference ($p = 0.07$)
Silva et al. [69]	parallel group design<br>ME: 12, 11 (m,f) / 9.5 ± 1.0<br>EE: 12, 11 (m,f) / 9.2 ± 1.0<br>Con: 11, 12 (m,f) / 9.5 ± 1.0<br>children with persistent moderate asthma, active (no regular exercise training program)	ME: 09:00<br>EE: 14:30<br>F: 2x/week<br>I: 5 min walking, 10-15 min running, rope skipping, body lifting movements, stepping on a bench, sit-ups, training on a bar, individual and team games, postural exercise + respiratory exercises: 45 min on dry land / 45 min in swimming pool<br>4 months	spirometric tests [Koko spirometer]<br>nine-minute running test<br>test time not reported	FEV1 (L)<br>FEV1 (%)<br>nine-minute running distance (m)	ME: 1.73 ± 0.29 / 1.81 ± 0.24<br>EE: 1.74 ± 0.38 / 1.80 ± 0.34<br>Con: 1.68 ± 0.34 / 1.76 ± 0.34<br>ME: 85 ± 14 / 86 ± 10<br>EE: 87 ± 14 / 88 ± 14<br>Con: 87 ± 14 / 85 ± 14<br>ME: 1344 ± 144 / 1592 ± 96<br>EE: 1327 ± 144 / 1489 ± 144<br>Con: 1310 ± 96 / 1313 ± 144	$p > 0.05$<br>$p > 0.05$<br>$p > 0.05$

Teo et al. [72,73]	parallel group design<br>ME: 9,11 (m,f) / 57 ± 5<br>EE: 8,12 (m,f) / 51 ± 13<br>overweight, inactive (activity < 150 min/week)<br>subgroup of T2D with oral hypoglycemic medications (ME: 10, EE: 10), BMI ≥ 27 kg/m^2<br>dropout rate: 0%	ME: 08:00-10:00<br>EE: 17:00-19:00<br>F: 3x/week<br>I: 60-70% VO_2peak<br>T: 30 min<br>T: treadmill walking<br>F: 3x/week<br>I: 3 x 12-18 reps at 45-55% 1RM<br>T: leg press, bench press, military press, lateral pulldown<br>12 weeks	blood analysis (venous blood sample)	HbA1c (%)	ME [all]: 6.85 ± 1.23 / 6.58 ± 1.1<br>EE [all]: 6.79 ± 1.66 / 6.54 ± 1.46<br>ME [T2D]: 7.91 ± 0.74 / 7.34 ± 0.81<br>EE [T2D]: 8.04 ± 1.44 / 7.64 ± 1.35	Group effect all: $p = 0.90$<br>Time effect all: $p < 0.01$<br>Group effect T2D: $p = 0.79$<br>Time effect T2D: $p < 0.01$
				fasting glucose (mmol/L)	ME [all]: 7.68 ± 1.70 / 6.78 ± 1.45<br>EE [all]: 8.28 ± 3.72 / 7.10 ± 2.41<br>ME [T2D]: 9.02 ± 1.33 / 7.76 ± 1.33<br>EE [T2D]: 10.32 ± 4.33 / 8.52 ± 2.69	Group effect all: $p = 0.55$<br>Time effect all: $p < 0.01$<br>Group effect T2D: $p = 0.39$<br>Time effect T2D: $p < 0.01$
				fasting insulin (pmol/L)	ME [all]: 88.3 ± 33.9 / 64.4 ± 23.4<br>EE [all]: 81.0 ± 29.7 / 58.6 ± 22.1<br>ME [T2D]: 81.7 ± 27.9 / 62.6 ± 21.5<br>EE [T2D]: 85.3 ± 24.6 / 64.4 ± 18.9	Group effect all: $p = 0.40$<br>Time effect all: $p < 0.01$<br>Group effect T2D: $p = 0.79$<br>Time effect T2D: $p < 0.01$
				HOMA2-IR	ME [all]: 1.78 ± 0.66 / 1.26 ± 0.46<br>EE [all]: 1.70 ± 0.71 / 1.16 ± 0.45<br>ME [T2D]: 1.74 ± 0.57 / 1.28 ± 0.44<br>EE [T2D]: 1.94 ± 0.70 / 1.35 ± 0.40	Group effect all: $p = 0.58$<br>Time effect all: $p < 0.01$<br>Group effect T2D: $p = 0.54$<br>Time effect T2D: $p < 0.01$
				fructosamine (µmol/L)	ME [all]: 262.5 ± 45.2 / 227.9 ± 77.8<br>EE [all]: 259.8 ± 53.6 / 230.2 ± 61.9<br>ME [T2D]: 286.5 ± 44.6 / 235.2 ± 108.5<br>EE [T2D]: 287.9 ± 57.7 / 267.9 ± 65.4	Group effect all: $p = 0.99$<br>Time effect all: $p < 0.01$<br>Group effect T2D: $p = 0.50$<br>Time effect T2D: $p = 0.09$
			dualenergy x-ray absorptiometry [Hologic Discovery W]	body fat (kg)	ME [al|: 27.4 ± 8.0 / 25.0 ± 8.0<br>EE [all]: 28.8 ± 7.4 / 26.3 ± 7.7	Group effect all: $p = 0.48$<br>Time effect all: $p < 0.01$
			step-incremental test (Bruce protocol) [treadmill]<br>test time for all groups: 07:00-07:30	VO_2peak (mL/min/kg)	ME [all]: 4.0 ± 1.7°<br>EE [all]: 4.1 ± 4.6°	Group effect all: $p = 0.89$<br>Time effect all: $p < 0.01$<br>°*differences from post to pre-measurement reported only*

Abbreviations: m, male; f, female; ME, morning exercise; EE, evening exercise; Con, control group; BMI, body mass index; SBP, systolic blood pressure; DBP, diastolic blood pressure; T2D, type 2 diabetes; HR, heart rate; VT, ventilatory threshold; VO_2max, maximal oxygen consumption; VO_2peak, peak oxygen consumption; RM, repetition maximum; HIIT, high intensity interval training; SJ, squat jump; CMJ, countermovement jump; HDL, high-density lipoprotein; LDL, low-density lipoprotein; sdLDL, small-dense low-density lipoprotein; TC, total cholesterol; PTH, parathyroid hormone; TSH, thyroid stimulating hormone; hsCRP, high-sensitivity C-reactive protein; MVC, maximum voluntary contraction; MVCI, maximum voluntary isometric contraction; MVC, maximum voluntary contraction; MVF, maximal voluntary force; MRFD, maximal rate of force development; FEV1, forced expiratory volume in one second; HbA1c, hemoglobin A1c.

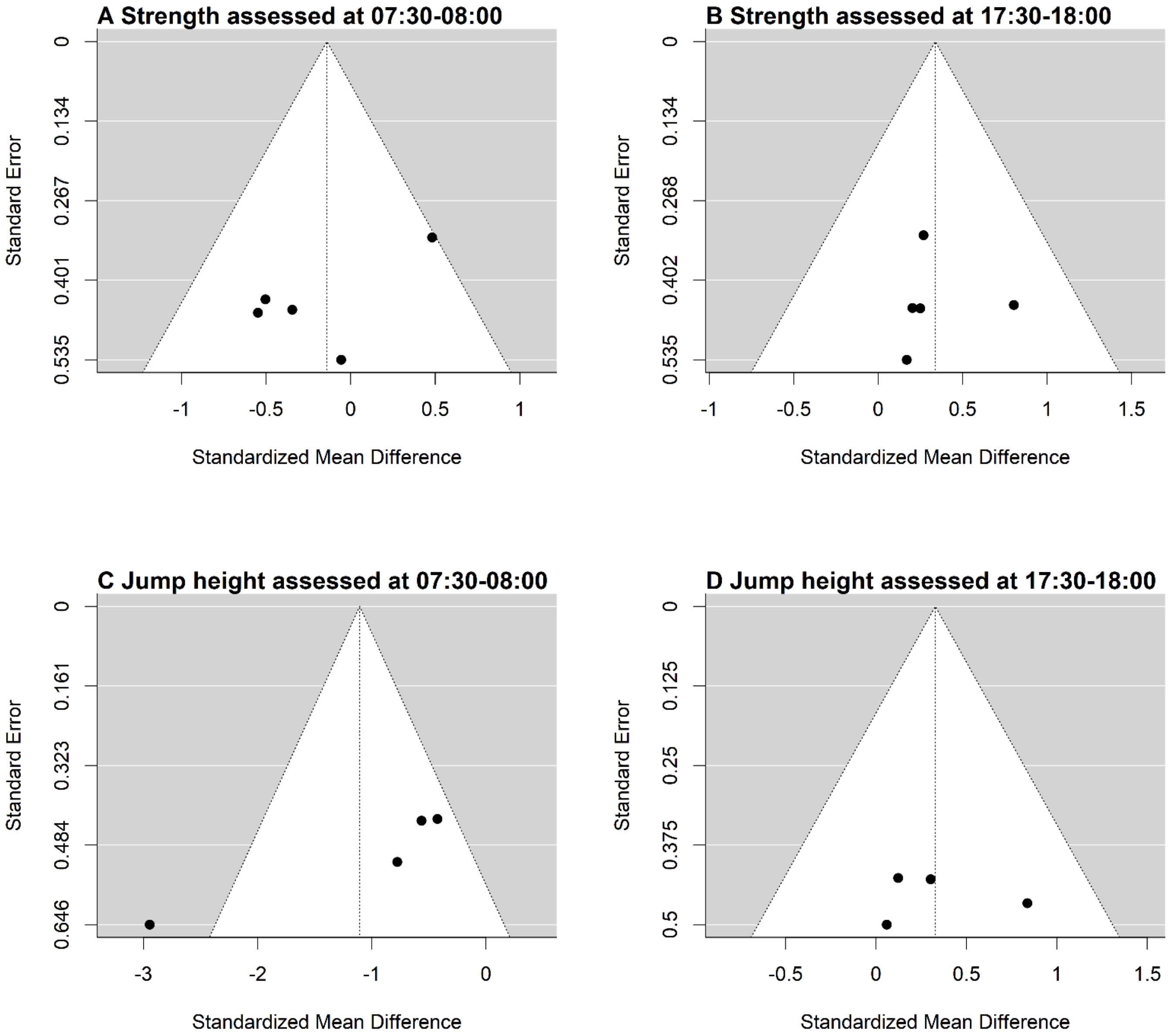

Supplemental Figure S1: Funnel plot for studies included in the meta-analysis.
